# Immunomodulatory Nanoparticles Enable Combination Therapies To Enhance Disease Prevention and Flare Control in Rheumatoid Arthritis

**DOI:** 10.1021/acscentsci.5c00723

**Published:** 2025-08-06

**Authors:** Wade T. Johnson, Elizabeth L. Wilkinson, Neha Iyer, Maksim Dolmat, Miriam Bollmann, Nour Dada, Xiaofu Wei, Shen Yang, Tiffany Zhang, Grace Yoo, Marianne Bernardo, Madison Price, Elizabeth Frame, Mariko Ishimori, Jon T. Giles, Wei Wang, Mattias N.D. Svensson, Nunzio Bottini, Nisarg J. Shah

**Affiliations:** † Department of Chemical and Nano Engineering, 8784 University of California San Diego, La Jolla, California 92093, United States; ‡ Department of Rheumatology and Inflammation Research, 3570 University of Gothenburg, Gothenburg 41346, Sweden; § Department of Chemistry and Biochemistry, University of California San Diego, La Jolla, California 92093, United States; ∥ Kao Autoimmunity Institute and Division of Rheumatology, 5149 Cedars-Sinai Medical Center, Los Angeles, California 90048, United States; ⊥ Department of Cellular and Molecular Medicine, University of California San Diego, La Jolla, California 92093, United States

## Abstract

Disease-modifying antirheumatic drugs (DMARDs) have greatly improved the treatment of rheumatoid arthritis (RA), but strategies to prevent disease onset and recurring flares remain limited. While abatacept (CTLA-4 IgG) can delay RA onset and corticosteroids are used for flare control, the benefit is temporary. We report that combining standard-of-care treatments with a locally administered immunomodulatory agent, termed Agg-CLNP, enhances both disease prevention and flare mitigation. Agg-CLNP consists of polymer nanoparticles conjugated with an immunodominant aggrecan peptide and encapsulate calcitriol. These nanoparticles are optimized for uptake by dendritic cells (DC) in lymph nodes proximal to arthritic joints. *In vitro*, Agg-CLNP suppressed costimulatory molecules and HLA class II (HLA-2) expression and upregulated CTLA-4 in human monocyte-derived DC from healthy and RA donors. In SKG mice, a T cell-driven RA model, Agg-CLNP combined with CTLA-4 IgG synergistically delayed disease onset and reduced severity. In a dexamethasone (Dex) withdrawal flare model, post-Dex Agg-CLNP treatment reduced flare severity and preserved a regulatory phenotype in DC, while suppressing local pathogenic T_H_17 cells. Next generation RNA sequencing of lymph node DC revealed *Ctla4* upregulation and changes in other immunomodulatory genes linked to flare prevention. These findings highlight Agg-CLNP as a potential therapeutic strategy to address critical unmet needs in RA management.

## Graphical abstract

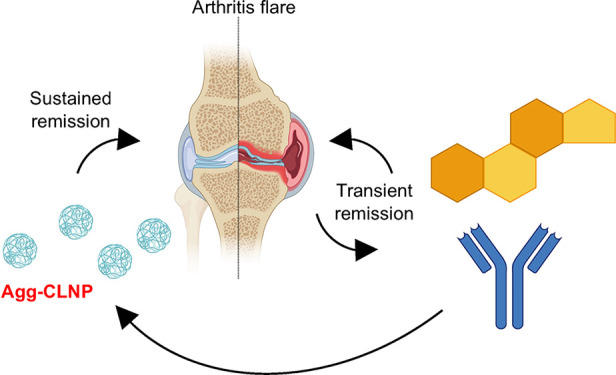

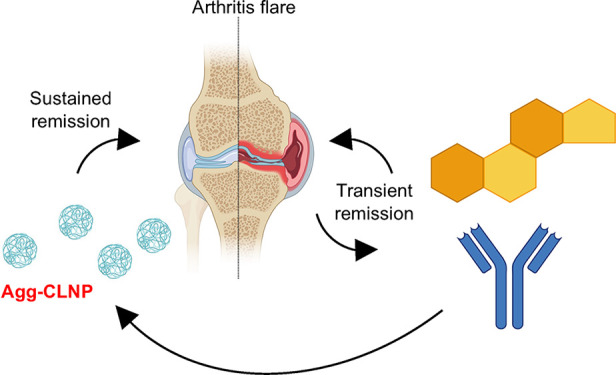

## Introduction

Rheumatoid arthritis (RA) is a chronic autoimmune disorder characterized by persistent joint inflammation, often resulting in progressive cartilage and bone damage.^1^ Disease-modifying antirheumatic drugs (DMARDs) have notably advanced RA management and more than 50% of patients achieve the target for treatment (remission or low disease activity [LDA]) using the current regimens.^2^ However, two important areas of unmet needs in RA are (i) preventing onset of disease in patients who are at risk because of the presence of anticitrullinated peptide antibodies (so-called pre-RA) and/or have a genetic predisposition for RA and (ii) sustaining the treatment target without temporary and unpredictable disease exacerbations, also known as flares.^3–5^

While the mechanisms of RA initiation and flare pathogenesis are complex, antigen-presenting cells, particularly dendritic cells (DC), play critical roles in initiating and perpetuating autoimmune inflammation.^6,7^ DC are activated in response to inflammatory stimuli, such as toll-like receptor ligands, and pro-inflammatory cytokines present within the synovial microenvironment.^8–11^ Activated autoantigen-presenting DC in joint-draining lymph nodes can prime autoreactive T cell responses in RA.^6^ These activated T cells can further infiltrate the inflamed synovial tissues, driving cytokine release, enhancing immune cell recruitment, and promoting local inflammation.^12,13^ Additionally, DC infiltrate synovial tissues, contributing directly to local inflammation through cytokine production and immune cell activation.^19–22^

Therapeutic strategies that shift DC toward an immunoregulatory phenotype are of substantial clinical interest. Abatacept, a biologic DMARD consisting of the extracellular domain of cytotoxic T-lymphocyte associated protein 4 (CTLA-4) fused to the fragment crystallizable (Fc) region of immunoglobulin G (CTLA-4 IgG), is a prime example of a DC immunomodulating agent and acts through competitive inhibition of CD80 and CD86 interactions with CD28 on T cells, effectively blocking DC-mediated costimulation required for T cell activation and proliferation.^14^ Abatacept treatment has been shown to delay disease onset for up to 18 months in the “Abatacept Reversing subclinical Inflammation as measured by MRI in ACPA positive Arthralgia” (ARIAA) trial^15^ and for up to 24 months in the “Arthritis Prevention In the Pre-clinical Phase of Rheumatoid Arthritis with Abatacept” (APIPPRA) trial.^16^ However, most patients still progressed to RA post-treatment. Moreover, achieving effective DC immunomodulation during active disease poses considerable challenges.^17–19^ While abatacept reduces disease activity in patients with active RA, it alone is insufficient to achieve flare-free remission in a large fraction of patients.^20–23^ In contrast to the above-mentioned clinical trials, flare-specific treatments have yet to be developed and trialed.^24^ Anti-inflammatory agents, mainly corticosteroids, are commonly employed and effective for rapid symptomatic relief during flares due to their potent anti-inflammatory properties.^25^ However, corticosteroids are often insufficient in preventing flare recurrence and the consequent structural joint deterioration, which tends to be proportional to the number and frequency of flares.^26–28^ In addition, repeated use of corticosteroids for flare control is associated with significant long-term side effects, including osteoporosis, hypertension, diabetes, and increased susceptibility to infections.^29^

In this manuscript, we report on the effectiveness of a polymer nanoparticle-based disease modifying agent, termed Agg-CLNP, to potentially augment the prevention of disease onset and flare control. Building on our prior work,^30^ here we elucidate the mechanism of action underlying the disease controlling effect of Agg-CLNP, optimize its therapeutic efficacy, and validate its potential as a novel complementary therapeutic strategy for RA disease and flare prevention. Agg-CLNP were formulated with a biodegradable poly­(lactic-*co*-glycolic acid)-poly­(ethylene glycol) (PLGA–PEG) copolymer functionalized with maleimide for conjugation of joint-relevant N-terminal cysteine-modified aggrecan antigen (Agg) by thiol-Michael addition. Agg is a shared immunodominant epitope between the BALB/c mouse major histocompatibility complex 2 (MHC2) I-A^d^ and the human leukocyte antigen (HLA)-DR4, which is a well-known strong risk factor for RA.^31,32^ Agg-CLNP encapsulated calcitriol, a known immunoregulator of DC and the active form of vitamin D3.^33,34^ Agg-CLNP were formulated to achieve a size <100 nm in diameter with a low polydispersity index, a size which is suited for passive lymphatic drainage. Agg-CLNP demonstrated robust DC immunomodulation *in vitro* in monocyte-derived DC sourced from healthy human and RA patient blood, reducing the expression of costimulatory molecules and HLA-2 and upregulating CTLA-4. In the T cell-driven SKG mouse model of RA, combination CTLA-4 IgG and Agg-CLNP significantly enhanced disease onset in prearthritic SKG mice compared to abatacept alone. In a dexamethasone (Dex) withdrawal flare model in SKG mice, Agg-CLNP reduced recurrent flare severity when administered. These effects were associated with lymph node DC characterized by the upregulation of immunomodulatory markers as assessed by flow cytometry and next generation bulk RNaseq. These findings highlight Agg-CLNP as a potential clinically relevant therapeutic strategy to address critical unmet needs in RA management.

## Results

### Agg-CLNP Has Low Polydispersity Index and Modulates SKG Dendritic Cells

Nanoparticles were formed by nanoprecipitation of an aggrecan peptide conjugated to a PEGylated polymer of PLGA and encapsulated calcitriol (Agg-CLNP). Agg-CLNP were sterile filtered and stored in 10 wt % sucrose at −20 °C (Figure 1a). Three batches of Agg-CLNP were synthesized and characterized. Agg-CLNP had an average z-avg of 70 nm with an average polydispersity index (PDI) of 0.08 (Figure 1b). Individual batches of Agg-CLNP possessed z-avgs of 74, 68, and 68 nm with PDIs of 0.06, 0.09, and 0.08 respectively (Table S1). The average calcitriol concentration in Agg-CLNP was 272 ng/mL, corresponding to a 21% encapsulation efficiency (*ee*), with individual batches at 267 ng/mL, 273 ng/mL, and 277 ng/mL with associated *ee* at 20.5%, 20.9%, and 21.2% (Table S2). Agg-CLNP were analyzed over the course of a month after freezing to confirm maintenance of low PDI and calcitriol content. Z-avg and PDI of Agg-CLNP were 69.6 nm and 0.005, 76.8 nm and 0.19, 68.4 nm and 0.03, and 75.6 nm and 0.08 at days 7, 14, 21, and 28 respectively (Figure 1c). Calcitriol concentration was quantitatively maintained over the course of 28 days in the formulation and remained stable (Figure 1d). To determine calcitriol release into the plasma, 22 μg of Agg-CLNP was injected intramuscularly (i.m.) into each biceps femoris of SKG mice, and plasma was analyzed by enzyme-linked immunosorbent assay (ELISA) 1 and 24 h post injection. Calcitriol concentration in the plasma was 23.0 ± 17.8 pg/mL and 16.1 ± 1.9 pg/mL at 1 and 24 h respectively, similar to untreated mice (14.2 ± 7.9 pg/mL) (Figure 1e). Bone marrow derived dendritic cells (BMDC) were cultured *in vitro* with Agg-CLNP dose matched to 1 nM calcitriol. After overnight stimulation with lipopolysaccharide (LPS), BMDC were analyzed by flow cytometry to quantify the expression of costimulatory molecules (CD80 and CD86) and MHC2. BMDC were identified as live CD45^+^CD11b^+^CD11c^+^ cells (Figure S1). Relative to LPS only treated BMDC, Agg-CLNP significantly reduced the fraction of CD80^hi+^ (Figure S2a), CD86^hi+^ (Figure S2b), and MHC2^hi+^ BMDC (Figure S2c).

**Figure 1:**
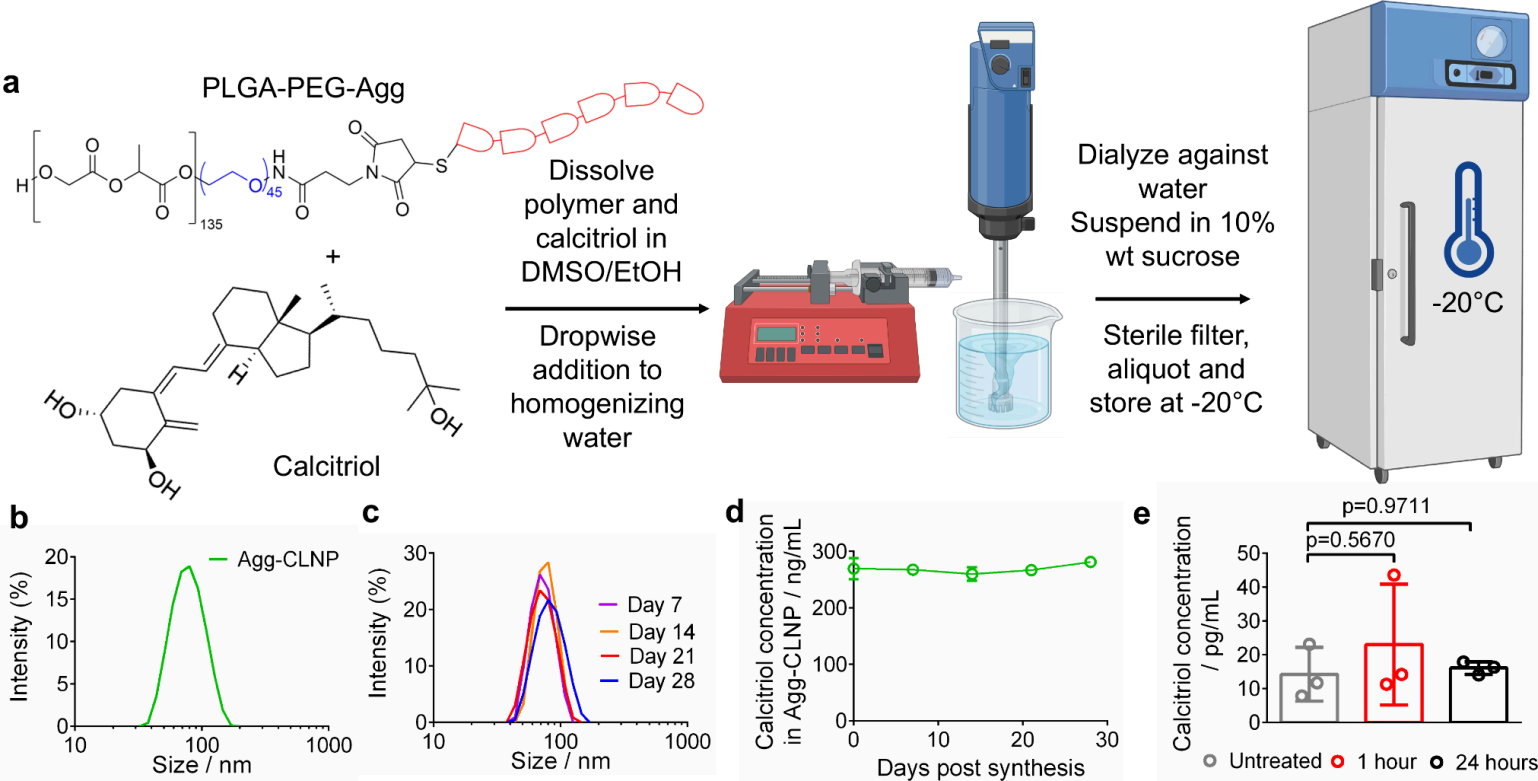
Synthesis and characterization of Agg-CLNP formulation. (a) Experimental schematic and chemical structure of aggrecan peptide conjugated calcitriol loaded nanoparticle synthesis. (b) Dynamic light scattering by intensity graph of Agg-CLNP formulation. (c) Dynamic light scattering by intensity plots of Agg-CLNP 7, 14, 21, and 28 days post synthesis. (d) Calcitriol content in Agg-CLNP 0, 7, 14, 21, and 28 days post synthesis as assessed by HPLC. (e) Calcitriol concentration in plasma at indicated time points and in untreated mice. Data in (d) are means ± SD of three technical replicates and in (e) are means ± SD of three experimental replicates. Statistical analyses in (e) were performed using ANOVA with Tukey’s multiple comparison test. Schematic in (a) was composed using BioRender and ChemDraw.

### Agg-CLNP Modulates Dendritic Cells Derived from Healthy Human Donor Blood

Human dendritic cells differentiated from monocytes (MDC) isolated from peripheral blood mononuclear cells were cultured *in vitro* with calcitriol (5 nM), dexamethasone (dex, 1 μM), Agg-CLNP (5 nM calcitriol dose matched), or a combination of dex and Agg-CLNP (at same doses previously listed) (Figure 2a). On day 0, the monocytes were plated at 1,000,000 cells/mL in a 96-well flat bottom tissue culture treated plate. On day 3, the media was refreshed. On day 4, calcitriol, dexamethasone, or Agg-CLNP were added (only dexamethasone was added on day 4 for the dex+Agg-CLNP group). On day 5, LPS was added to achieve 500 ng/mL LPS to all wells. Agg-CLNP was also added on day 5 to the dex+Agg-CLNP group. After a 48-h stimulation with LPS, MDC were analyzed by flow cytometry to quantify the expression of costimulatory molecules (CD40, CD80 and CD86), HLA-2, and CTLA-4 on day 7. MDC were identified as live CD11c^+^ cells (Figure S3). Dexamethasone, Agg-CLNP and dex+Agg-CLNP treatments significantly reduced the concentration of IL-6 (Figure 2b) and TNF (Figure 2c) in the culture supernatant relative to LPS only stimulated MDC. Relative to LPS only treated MDC, all treatments significantly reduced the fraction of CD40^hi+^ (Figure 2d), CD80^hi+^ (Figure 2e), CD86^hi+^ (Figure 2f), and HLA-2^hi+^ (Figure 2g). Only calcitriol and Agg-CLNP treatments significantly increased the fraction of CTLA-4^+^ MDC relative to LPS only treated MDC (70.2% ± 7.6% calcitriol, 88.8% ± 4.4% Agg-CLNP vs 14.9% ± 7.0% LPS only) (Figure 2h).

**Figure 2:**
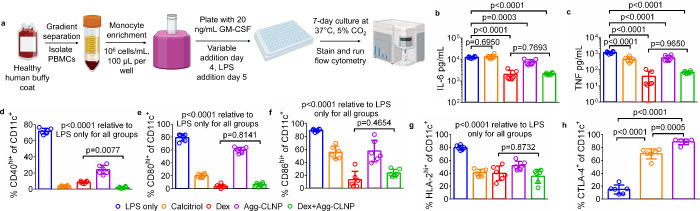
Agg-CLNP imparts an immunomodulatory phenotype on healthy human MDC *in vitro*. (a) Experimental schematic of MDC culture. (b and c) Proinflammatory cytokine concentrations in cell culture as measured by (b) IL-6 and (c) TNF. (d–h) Costimulatory molecules (CD40, CD80, and CD86), HLA-2, and CTLA-4 positivity on CD11c^+^ MDC after culture with LPS only, free calcitriol, free dexamethasone, Agg-CLNP and a combination of dexamethasone with Agg-CLNP as measured by (d) CD40, (e) CD80, (f) CD86, (g) HLA-2, and (h) CTLA-4. Data in (b–h) are means ± SD of six technical replicates from representative experiments. Statistical analyses in (b–h) were performed using ANOVA with Tukey’s multiple comparison test. Schematic in (a) was composed using BioRender.

### Agg-CLNP Modulates Dendritic Cells Derived from RA Patient Donor Blood

Human dendritic cells differentiated from monocytes (MDC) isolated from peripheral blood mononuclear cells of RA patient donors were cultured *in vitro* with Agg-CLNP (5 nM calcitriol dose) (Figure 3a). On day 0, the monocytes were plated at 1,000,000 cells/mL in a 96-well flat bottom tissue culture treated plate. On day 3, the media was refreshed. On day 4, Agg-CLNP was added. On day 5, LPS was added to achieve 500 ng/mL LPS to all wells. After a 48-h stimulation with LPS, MDC were analyzed by flow cytometry to quantify the expression of costimulatory molecules (CD40, CD80 and CD86), CTLA-4 and HLA-2 on day 7. MDC were identified as live CD11c^+^ cells (Figure S4). Agg-CLNP significantly reduced the fraction of CD40^hi+^ (Figure 3b), CD80^hi+^ (Figure 3c) MDC relative to LPS only treatment. In aggregate, Agg-CLNP significantly reduced the fraction of CD86^hi+^ (Figure 3d) MDC relative to LPS only treatment. In all donors, Agg-CLNP also significantly reduced the fraction of HLA-2^hi+^ MDC relative to LPS only treated MDC (Figure 3e). In aggregated donor data, Agg-CLNP significantly increased the fraction of CTLA-4^+^ DC relative to LPS only treated DC (Figure 3f). Data disaggregated by donor are provided in Figure S5. Donor characteristics are provided in Table S3.

**Figure 3:**
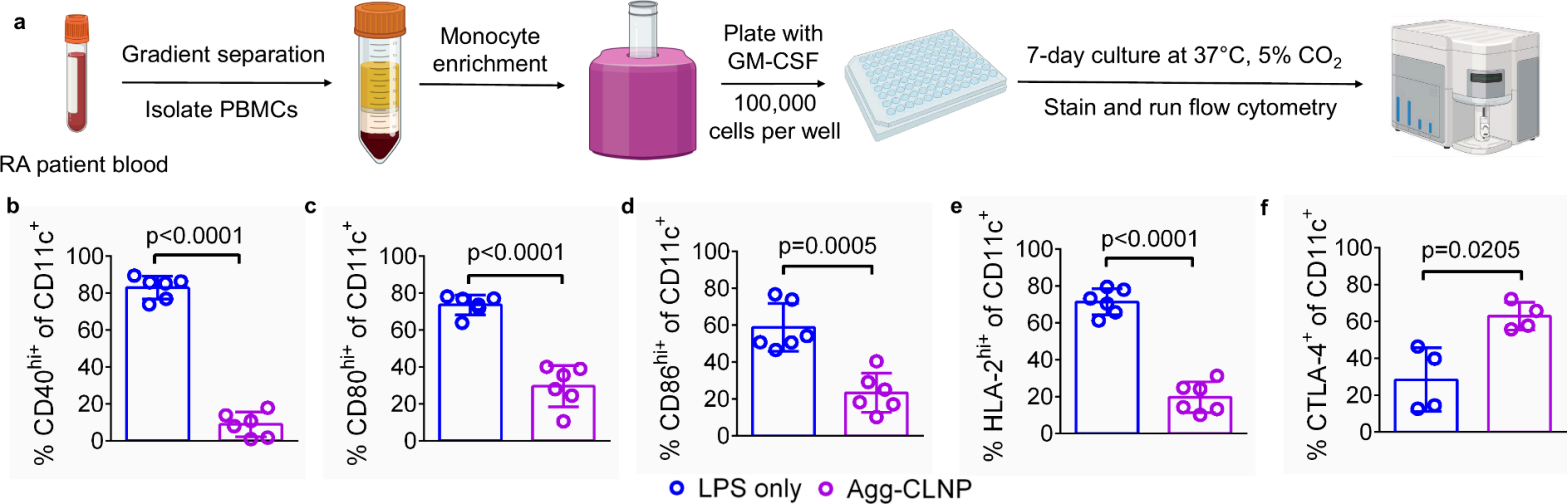
Agg-CLNP imparts an immunomodulatory phenotype on RA patient human MDC *in vitro*. (a) Experimental schematic of MDC culture. (b–f) Costimulatory molecules (CD40, CD80, and CD86), HLA-2, and CTLA-4 positivity on CD11c^+^ MDC after culture with LPS only or Agg-CLNP as measured by (b) CD40, (c) CD80, (d) CD86, (e) HLA-2, and (f) CTLA-4. Data in (b–f) are means ± SD, where each data point represents the mean of a single donor. Statistical analyses in (b–f) were performed with unpaired Students *t*-test with Welsch’s correction. Schematic in (a) was composed using BioRender.

### CTLA-4 IgG in Combination with Agg-CLNP Reduces Arthritis Onset and Severity in SKG Mice

BALB/c SKG mice were chosen to model RA in these studies as the mice develop inflammatory polyarthritis characterized by rheumatoid factor (RF), anticitrullinated protein antibodies (ACPA) and symmetric affection of small joints.^35,36^ SKG arthritis is driven by arthritogenic T cells which can be synchronized by intraperitoneal (i.p.) injection of mannan, a fungal polysaccharide. In all SKG experiments, arthritis was synchronized by mannan injection on day 0. Agg-CLNP were administered either prophylactically (3 days of daily injections before arthritis synchronization) or as treatment (injections on days 14–16 post arthritis synchronization) in the SKG model of arthritis by i.m. injections into each biceps femoris (22 μg/day). When administered as a prophylactic, Agg-CLNP treated mice consistently had a lower clinical score relative to untreated mice, resulting in a significant difference of 1.2 ± 0.38 Agg-CLNP treated vs 3.0 ± 0.37 untreated at day 14 (Figure S6a). However, in established disease Agg-CLNP alone was ineffective at modulating arthritis severity (Figure S6b). We therefore sought to assess if the disease-preventative effect of Agg-CLNP might enhance the effect of CTLA-4 IgG. CTLA-4 IgG, human IgG, and Agg-CLNP were administered on days 0–2 (250 μg/day for CTLA-4 IgG and human IgG via i.p. injection, 22 μg/day for Agg-CLNP via i.m. into each biceps femoris) in tandem with arthritis synchronization with i.p. mannan injection on day 0 (Figure 4a). Clinical scores (Figure 4b) and ankle thickness (Figure 4c) were measured for 14 days. At the end point, T cells in the lymph nodes were analyzed by flow cytometry and ankles were fixed and processed for histology. The clinical score of CTLA-4 IgG+Agg-CLNP treated mice was 0.58 ± 0.26 at day 14, significantly lower than those of untreated mice, human IgG treated mice, and CTLA-4 IgG only treated mice (2.6 ± 0.38) at the same time point. The change in ankle thickness of CTLA-4 IgG+Agg-CLNP treated mice was 0.23 ± 0.06 mm at day 14, significantly lower than the aforementioned comparison groups at the same time point. Kaplan–Meier curves of arthritis incidence (score >0), low disease score (score >0.5) and high disease score (score >1) showed that all untreated mice achieved a score greater than 0 by day 3, all human IgG and CTLA-4 IgG mice achieved a score greater than 0 by day 7, and one mouse did not develop arthritis in the CTLA-4 IgG+Agg-CLNP group (Figure 4d). All untreated and human IgG mice achieved a score greater than 0.5 by day 7, all CTLA-4 IgG mice achieved a score greater than 0.5 by day 14, and three mice did not achieve a score greater than 0.5 in the CTLA-4 IgG+Agg-CLNP group, a significant difference (Figure 4e). All untreated and human IgG mice achieved a score greater than 1 by day 7, all CTLA-4 IgG mice achieved a score greater than 1 by day 14, and five mice did not achieve a score greater than 1 in the CTLA-4 IgG+Agg-CLNP group, a significant difference (Figure 4f).

**Figure 4:**
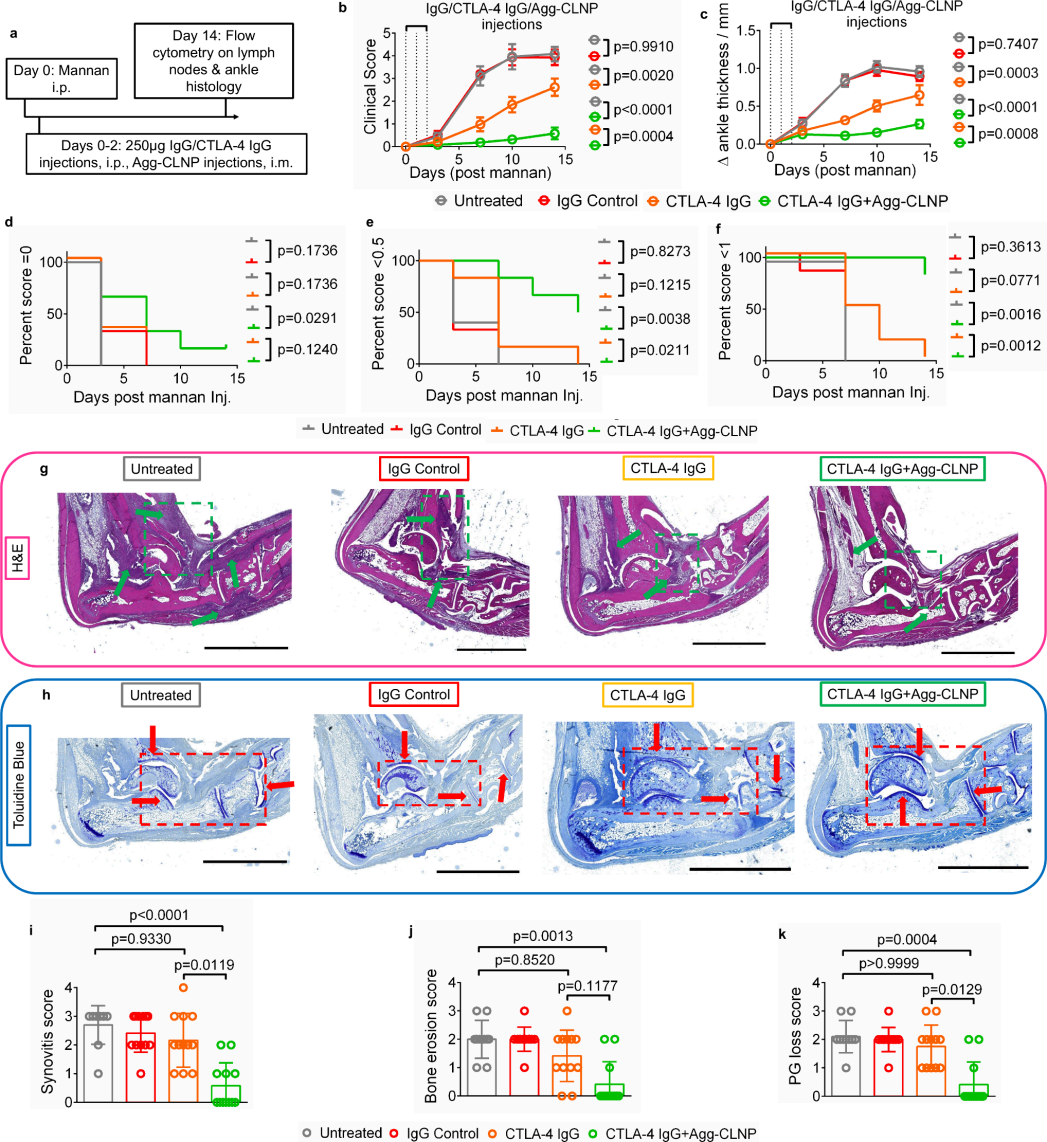
Agg-CLNP in combination with CTLA-4 IgG reduces RA onset and severity in SKG mice. (a) Timeline of experimental procedure. (b) Clinical scores of untreated mice (*n* = 5) and mice treated with bolus IgG (250 μg/day on days 0–2, *n* = 6), bolus CTLA-4 IgG (250 μg/day on days 0–2, *n* = 6) or bolus CTLA-4 IgG in tandem with Agg-CLNP (22 μg Agg-CLNP/biceps femoris/day, on days 0–2, *n* = 6). (c) Ankle thickness deltas of the mice clinically scored in b. (d–f) Kaplan–Meier curves of arthritis incidence and severity as measured by (d) incidence, (e) low disease score, and (f) high disease score. (g) Representative H&E-stained ankle sections from untreated group (*n* = 10), IgG control group (*n* = 12), CTLA-4 IgG group (*n* = 12), and CTLA-4 IgG + Agg-CLNP group (*n* = 12). (h) Representative toluidine blue stained ankle sections from untreated group (*n* = 10), IgG control group (*n* = 12), CTLA-4 IgG group (*n* = 12), and CTLA-4 IgG + Agg-CLNP group (*n* = 12). (i–k) Histological scoring of mouse ankles as measured by (i) synovitis score, (j) bone erosion score, and (k) proteoglycan loss score. Data in (b) and (c) are means ± SEM. Data in (i–k) are means ± SD. Green arrows in (g) represent regions of synovitis and red arrows in (h) indicate cartilage on articulating surfaces. Green boxes in (g) and red boxes in (h) are regions of interest whose magnified images are provided in the Supporting Information. Statistical analyses performed using (b and c) two-way ANOVA, (d–f) log-rank Mantel–Cox, and (i–k) Kruskal–Wallis test. Scale bar in (g–h) is 2 mm.

Ankles were fixed, sectioned, and stained with either hematoxylin and eosin (H&E) (Figures 4g, S7, and S8) or toluidine blue (Figures 4h, S7, and S8). H&E-stained sections were blindly scored for synovitis and bone erosion, while toluidine blue sections were blindly scored for proteoglycan (PG) loss. CTLA-4 IgG+Agg-CLNP showed significantly lower synovitis (0.58 ± 0.79) compared to untreated, human IgG control, and CTLA-4 IgG only sections (Figure 4i). CTLA-4 IgG+Agg-CLNP showed significantly lower bone erosions (0.42 ± 0.79) compared to untreated and human IgG control sections, and lower bone erosions compared to CTLA-4 IgG only sections (1.4 ± 0.90) (Figure 4j). CTLA-4 IgG+Agg-CLNP showed significantly lower PG loss (0.42 ± 0.79) compared to untreated, human IgG control, and CTLA-4 IgG only sections (Figure 4k). Histopathological evaluations between untreated, human IgG and CTLA-4 IgG only treated mice were comparable.

Inguinal and popliteal lymph nodes were homogenized to obtain single cell suspensions and analyzed for the inducible T cell costimulator (ICOS) activation marker (Figure S9). CTLA-4 IgG+Agg-CLNP treatment significantly reduced the fraction of live CD4^+^ICOS^+^ T cells relative to untreated and IgG treated mice and reduced the fraction of CD4^+^ICOS^+^ cells relative to CTLA-4 IgG treated mice (Figure S10a). CTLA-4 IgG+Agg-CLNP treatment significantly reduced the number of CD4^+^ICOS^+^ T cells relative to untreated and IgG treated mice and reduced the fraction of CD4^+^ICOS^+^ T cells relative to CTLA-4 IgG treated mice (Figure S10b).

### Agg-CLNP Maintain a Less Inflammatory Profile of Dendritic Cells Post Dexamethasone in Lymph Nodes of SKG Mice

The efficacy of Agg-CLNP as a disease-preventative agent prompted an assessment of its effectiveness in mice that were previously arthritic but have accomplished a low disease state. We established a dexamethasone withdrawal flare model in which Agg-CLNP were administered after dexamethasone-induced low disease state in arthritic SKG. SKG mice were injected i.p. with mannan on day 0 to synchronize onset of arthritis. Dexamethasone was administered by daily i.p. injection on days 8–10 (125 μg/day) followed by daily i.m. Agg-CLNP injection on days 11–13 into each biceps femoris (22 μg/day) in a subset of mice (Figure 5a). Clinical scores were monitored for 14 days post mannan injection (Figure 5b). The clinical score of dexamethasone only treated mice was 0.37 ± 0.08 on day 11, lower than that of untreated mice at the same time point. Dexamethasone and Agg-CLNP combination treatment in mice maintained a clinical score of 0.7 ± 0.32 on day 14, significantly lower than that of untreated mice (4.38 ± 0.18) and dexamethasone only mice (2.85 ± 0.16) at the same time point. On days 11 and 14, a subset of mice was sacrificed, and the inguinal and popliteal lymph nodes were excised. Lymph nodes from two mouse cohorts were combined for flow cytometry analysis. The DC immunophenotype between untreated and dexamethasone only treated mice were compared on day 11, while the DC immunophenotype between all groups was compared on day 14. DC were identified as live CD45^+^CD11b^+^CD11c^+^ cells (Figure S11) and analyzed for expression of costimulatory molecules CD80 and CD86, and MHC2. Dexamethasone treated mice significantly lower CD80^hi+^ (Figure 5c), CD86^hi+^ (Figure 5d), and MHC2^hi+^ (Figure 5e) compared to untreated mice on day 11. In mice that were additionally administered Agg-CLNP, CD80^hi+^ (Figure 5f), CD86^hi+^ (Figure 5g), and MHC2^hi+^ (Figure 5h) expression was significantly lower compared to to untreated and dexamethasone only mice on day 14. All groups had similar CD45^+^ (Figure S12a), CD45^+^CD11b^+^ (Figure S12b), and CD45^+^CD11b^+^CD11c^+^ (Figure S12c) cell counts.

**Figure 5:**
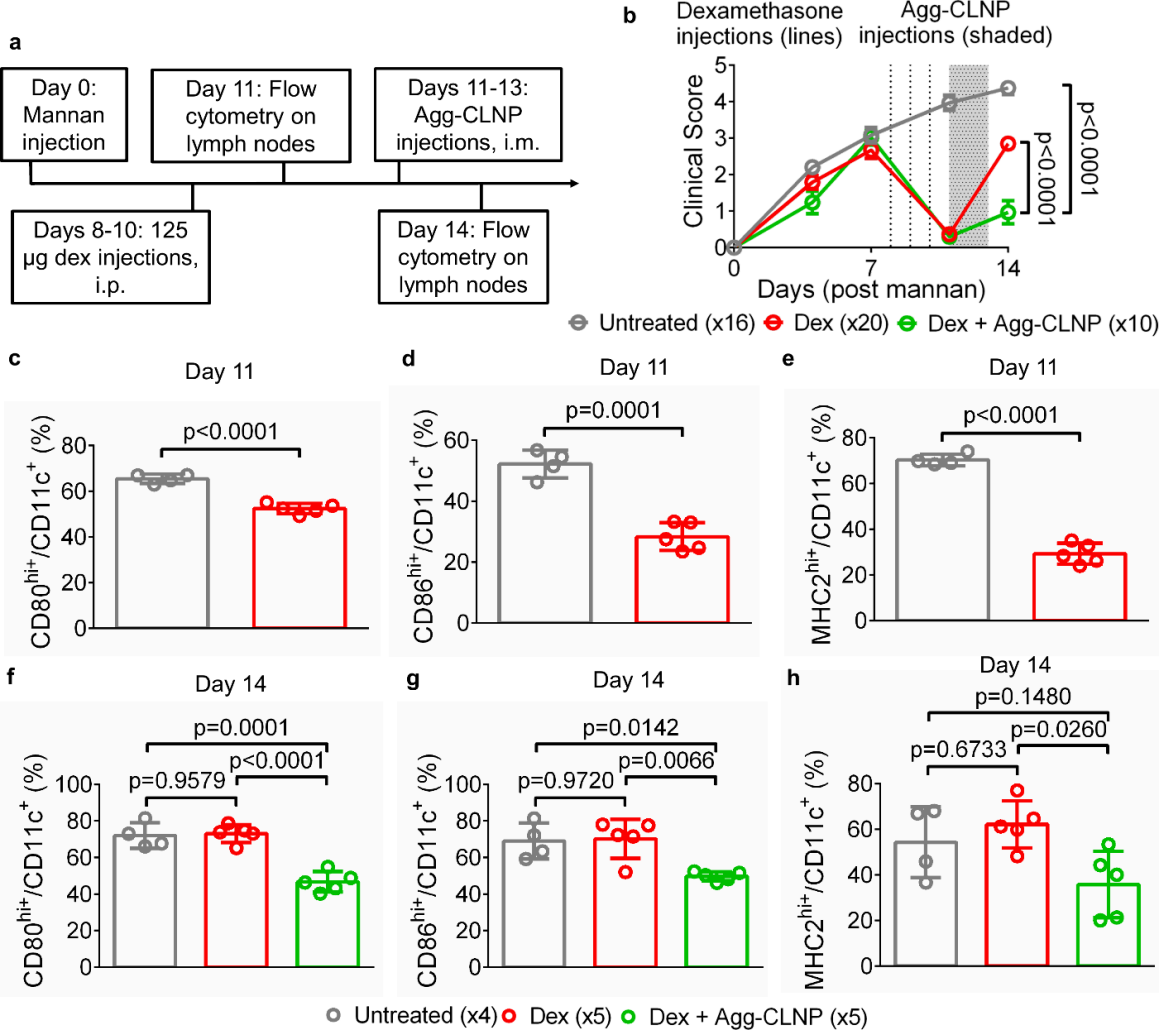
Agg-CLNP in combination with dexamethasone modulates DC phenotype in SKG mice. (a) Schematic and timeline of experimental procedure. (b) Clinical scores of untreated mice (*n* = 16) and mice treated with either bolus dexamethasone (125 μg/day, *n* = 20) or bolus dexamethasone followed by Agg-CLNP (22 μg Agg-CLNP/biceps femoris/day, *n* = 10). (c–e) Costimulatory molecules (CD80 and CD86) and MHC-2 CD11c^+^ DC from lymph nodes on day 11 as measured by (c) CD80, (d) CD86, and (e) MHC2. (f–h) Costimulatory molecules (CD80 and CD86) and MHC2 CD11c^+^ DC from lymph nodes on day 14 as measured by (f) CD80, (g) CD86, and (h) MHC2. Data in (b) are means ± SEM. Data in (c–h) are means ± SD. Statistical analyses in (b, f–h) were performed using ANOVA with Tukey’s multiple comparison test and in (c–e) were performed with unpaired Student’s *t*-test with Welsch’s correction. Statistical analysis of (b) consisted solely of day 14 clinical scores.

To assess the effectiveness of combination therapy upon coadministered of Agg-CLNP and dexamethasone, we injected SKG mice on days 8–10 with i.p. dexamethasone (125 μg/day) and i.m. Agg-CLNP (22 μg/day) after arthritis synchronization with mannan on day 0 (Figure S13a). Untreated mice and mice treated only with dexamethasone were included as controls. The clinical score of Agg-CLNP and dexamethasone combination treated mice was 1.25 ± 0.30 on day 14, significantly lower than that of untreated mice (3.56 ± 0.35) and dexamethasone only mice (2.77 ± 0.39) at the same time point (Figure S13b). However, DC from lymph node, analyzed for CD80 (Figure S13c), CD86 (Figure S13d), and MHC2 (Figure S13e) showed no significant differences.

To assess if low disease activity induced by dexamethasone affected CLNP uptake by DC, we conjugated cyanine 5 (Cy5)-PEG-thiol to the PLGA–PEG-MAL via maleimide–thiol click chemistry and formulated Cy5-CLNP following the same procedure to formulate Agg-CLNP. SKG mice were injected i.p. with mannan on day 0 to synchronize onset of arthritis. Dexamethasone was administered by daily i.p. injection on days 8–10 (125 μg/day) in a subset of mice followed by daily i.m. Cy5-CLNP injection on days 11–13 into each biceps femoris (22 μg/day) for all mice (Figure S14a). There were no significant differences in clinical score at day 14 between mice that received Cy5-CLNP (2.84 ± 0.37) and mice that received both dex and Cy5-CLNP (1.86 ± 0.49) (Figure S14b). Similar fraction of Cy5^+^ CD11c^+^ DC in the lymph nodes were observed in Cy5-CLNP- (70.0% ± 9.7%) and Dex+Cy5-CLNP-treated mice (67.9% ± 8.1%) (Figure S14c). These results show that dexamethasone does not affect uptake of CLNP by DC, and the Agg peptide is critical for flare modulation.

### Agg-CLNP Post Dexamethasone Locally Modulates TH17 in the Proximal Lymph Nodes and Paws

T_H_17 T cells are key mediators of arthritis in SKG mice. We sought to assess the effect of the aforementioned treatments on T_H_17 T cells. Dexamethasone was administered by daily i.p. injection on days 8–10 (125 μg/day) followed by daily i.m. Agg-CLNP injection on days 11–13 into each biceps femoris (22 μg/day) (Figure S15a). Subsequently, the spleen, popliteal and inguinal lymph nodes, and hind paws were harvested and stained for T_H_17 cells, which were identified as live CD45^+^CD4^+^IL-17^+^ cells (Figure S9). No differences were observed in the spleen between combination dexamethasone and Agg-CLNP and dexamethasone only treated mice (Figure S15b). In the combined inguinal and popliteal lymph nodes (Figure S15c) and the hind paws (Figure S15d), combination dexamethasone and Agg-CLNP treatment significantly reduced T_H_17 T cell counts compared to dexamethasone only treated mice.

### Agg-CLNP Post Dexamethasone Modulates Subsequent Arthritis Flares in SKG Mice

Corticosteroids are commonly used for treating flares and can provide transient symptomatic relief, but do not modify disease or prevent flare recurrence. We sought to test if Agg-CLNP might extend dexamethasone-induced low disease activity and modulate flare recurrence. Dexamethasone was administered by daily i.p. injection on days 8–10, 22–24, and 36–38 (25 μg/day) followed by daily i.m. Agg-CLNP injection on days 11–13, 25–27, and 39–41 into each biceps femoris (22 μg/day) (Figure 6a). Clinical scores (Figure 6b) and ankle thickness deltas (Figure 6c) were monitored for 42 days post mannan injection. The clinical scores of postdexamethasone Agg-CLNP treated mice were 1.1 ± 0.20, 1.8 ± 0.46, and 1.9 ± 0.46 at days 14, 28, and 42 respectively, significantly lower than those of untreated mice (4.1 ± 0.45, 3.9 ± 0.48 and 4.0 ± 0.67) and dexamethasone only treated mice (2.2 ± 0.34, 3.4 ± 0.39 and 3.8 ± 0.40) at the same time points. The ankle thickness deltas of Agg-CLNP treated mice were 0.25 ± 0.04 mm, 0.45 ± 0.09 mm, and 0.38 ± 0.09 mm at days 14, 28, and 42 respectively, significantly lower than untreated mice (0.93 ± 0.15 mm, 0.99 ± 0.18 mm and 0.89 ± 0.15 mm), and dexamethasone only treated mice (0.52 ± 0.07 mm, 0.83 ± 0.07 mm and 0.76 ± 0.07 mm) at the same time points. Notably, dexamethasone alone was less effective at reducing disease activity upon repeated injections. The absolute clinical score change between days 7–11, 21–25, and 35–39 of dexamethasone only mice were 2.2, 1.5, and 0.82 respectively. On the other hand, post- dexamethasone Agg-CLNP treatment was more effective upon repeated injections. The absolute clinical score Δ between days 11–14, 25–28, and 39–42 of Agg-CLNP treated mice were 0.61, 0.59, and 0.55 respectively. Ankles were fixed, sectioned, and stained with either H&E (Figure 6d, S16, S17) or toluidine blue (Figures 6e, S16, and S17). H&E-stained sections were blindly scored for synovitis and bone erosion, while toluidine blue sections were blindly scored for proteoglycan loss. Dex+Agg-CLNP treatment significantly reduced synovitis (Figure 6f), bone erosions (Figure 6g), and proteoglycan (PG) loss (Figure 6h) compared to dexamethasone only treatment and no treatment.

**Figure 6:**
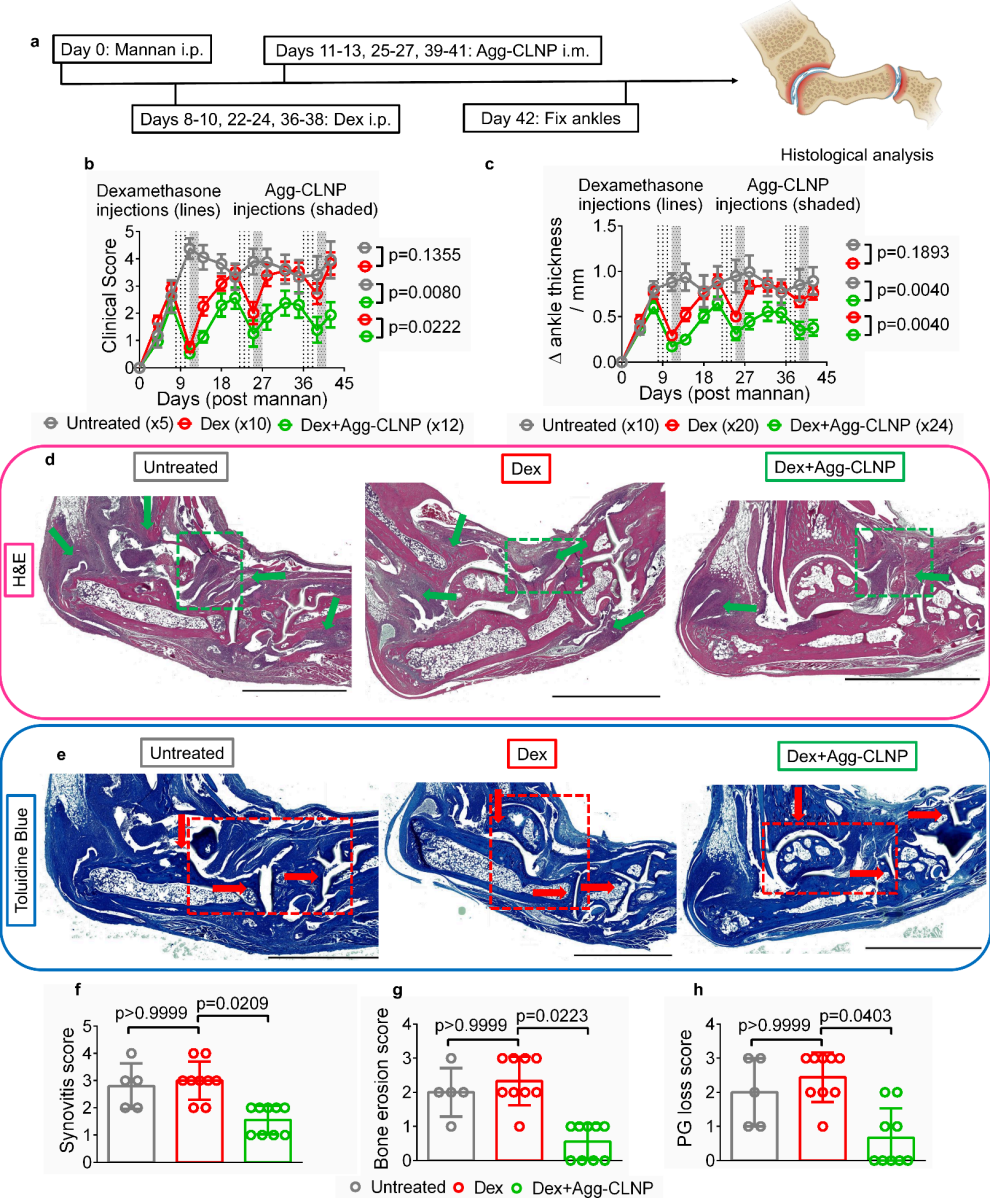
Agg-CLNP modulates flare in dexamethasone withdrawal in SKG mice. (a) Schematic and timeline of experimental procedure. (b) Clinical scores of untreated mice (*n* = 5) and mice treated with either bolus dexamethasone (25 μg/day on days 8–10, 22–24, and 36–38, *n* = 10) or bolus dexamethasone followed by Agg-CLNP (22 μg Agg-CLNP/biceps femoris/day, on days 11–13, 25–27, and 39–41, *n* = 12). (c) Ankle thickness deltas of the mice clinically scored in b. (d) Representative H&E-stained ankle sections from untreated group (*n* = 5), dex only group (*n* = 9), and dex+Agg-CLNP group (*n* = 9). (e) Representative toluidine blue stained ankle sections from untreated group (*n* = 5), dex only group (*n* = 9), and dex+Agg-CLNP group (*n* = 9). (f–h) Histological scoring of mouse ankles as measured by (f) synovitis score, (g) bone erosion score, and (h) proteoglycan loss score. Green arrows in (d) represent regions of synovitis and red arrows in (e) indicate cartilage on articulating surfaces. Green boxes in (d) and red boxes in (e) are regions of interest whose magnified images are provided in the Supporting Information. Data in (b) and (c) are means ± SEM. Data in (f–h) are means ± SD. Statistical analyses were performed using (b and c) two-way ANOVA and (f–h) Kruskal–Wallis test. Scale bar in (d) and (e) is 2 mm.

### Agg-CLNP Modulates RA-Relevant Genes in DC from Lymph Nodes of SKG Mice

While flow analysis performed in Figure 5 indicated Agg-CLNP maintained an immunomodulatory DC phenotype initially imparted by dexamethasone, we sought to identify other changes in DC immunomodulatory markers associated with Agg-CLNP. To analyze the transcriptomic profile of DC treated with Agg-CLNP post dexamethasone, we conducted a next generation bulk RNA-seq analysis of DC isolated from the lymph nodes of SKG mice following dexamethasone alone, or a combination of dexamethasone followed by Agg-CLNP. Dexamethasone was administered by daily i.p. injection on days 8–10 (125 μg/day) followed by daily i.m. Agg-CLNP injection on days 11–13 into each biceps femoris (22 μg/day) (Figure 7a). Clinical scores were monitored for 14 days post mannan injection (Figure 7b). The clinical score of dexamethasone+Agg-CLNP treated mice was 0.88 ± 0.27 at day 14, significantly lower than dexamethasone only treated mice (2.9 ± 0.25) at the same time point. On day 14, the mice were sacrificed, and the inguinal and popliteal lymph nodes were harvested. The lymph nodes from four mice per group were pooled and homogenized to obtain three experimental replicate cell suspensions per group and sequenced. A volcano plot of the differentially expressed genes (DEG) showed genes significantly upregulated in the dexamethasone+Agg-CLNP group relative to dexamethasone only in the upper right quadrant (p_adj_ ≤ 0.05, and log_2_ fold-change ≥ 1.25) and significantly downregulated in the dexamethasone+Agg-CLNP group relative to dexamethasone only group in the upper left quadrant (p_adj_ ≤ 0.05, and log_2_ fold-change ≤ −1.25) (Figure 7c). Potential RA-associated genes of interest included *Card14*, *Ccl22*, *Cd200*, *Ctla4*, and *Tsc22d3*. A dendrogram of the top 60 up- (in orange) and down- (in blue) regulated DEG showed all genes of interest are within this range (Figure 7d).

**Figure 7:**
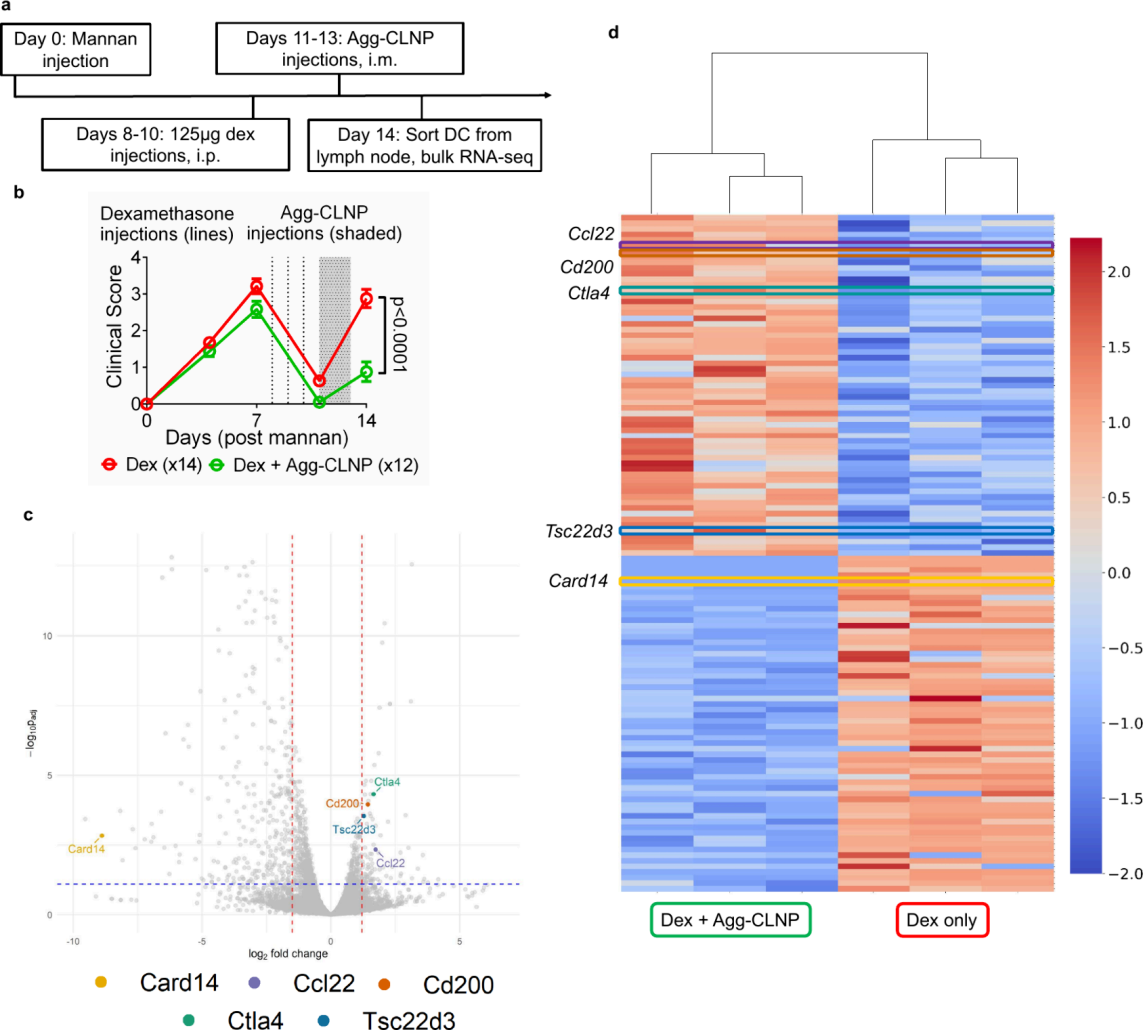
Agg-CLNP in combination with dexamethasone modulates RA relevant targets in DC from SKG lymph nodes. (a) Timeline of experimental procedure. (b) Clinical scores of mice treated with either bolus dexamethasone (125 μg/day, *n* = 12) or bolus dexamethasone followed by Agg-CLNP (22 μg Agg-CLNP/biceps femoris/day, *n* = 12). (c) Volcano plot with RA-associated genes indicated in the legend. (d) Dendrogram comparing the top 60 upregulated and top 60 downregulated DEG from bulk RNA-seq, *Ccl22*, *Cd200*, *Ctla4*, *Tsc22d3*, and *Card14* are highlighted. In (c), blue line represents a *p*_adj_ value ≤ 0.05 and red lines indicate a log_2_ fold-change ≥ 1.25 or ≤ −1.25. In (d), each column represents a replicate of the respective condition and data represents the signal across each gene ranked as *z*-scores using data across each row. Data in (b) are means ± SEM. Statistical analysis in (b) was performed by unpaired Student’s *t*-test with Welsh’s correction of day 14 clinical scores.

## Discussion

Preventing the onset of RA in at-risk patients and flares of disease in patients with established disease are high-priority unmet needs.^37^ Our approach developed and characterized Agg-CLNP for the modulation of DC, which are key antigen presenting cells in RA disease pathology.^12^ Formulation-optimized sterile Agg-CLNP had a consistent particle size and composition, low polydispersity, and were stable for at least one month under frozen conditions. We demonstrate that Agg-CLNP consistently modulate activation of mouse BMDC and human MDC from healthy and RA patients. In combination with CTLA-4 IgG, Agg-CLNP durably reduced arthritis onset and severity in SKG mice, significantly more than CTLA-4 IgG alone. To mimic RA flares in SKG mice, we developed a dexamethasone flare withdrawal model. Agg-CLNP administered post dexamethasone consistently mitigated arthritis flare and was associated with immunomodulatory DC in the draining lymph nodes with reduced T_H_17 pathogenic T cells. Bulk next generation RNA-seq of DC isolated from the lymph nodes of mice treated with Agg-CLNP, revealed potential genes associated with flare protective effects. Our results support the potential of Agg-CLNP as a RA preventive and flare control agent, which could also be combined with current standard-of-care RA therapies.

The concept of targeting DC for RA control has been an active area of research. DC-therapy clinical protocols include ex vivo generation and intradermal injection of tolerogenic DC after incubation with a mixture of RA autoantigens (Rheumavax).^38^ Another approach in RA uses intralymph node injection of tolerogenic DC pulsed with the heat shock protein (HSP)-derived B29 peptides.^39,40^ Our approach is aligned with nanoparticle-based tolerization strategies for APCs that are in preclinical^41–44^ and clinical development,^45^ including for RA.^46^ Nanoparticle-based DC-targeting has also been reported.^47^ By encapsulating therapeutic peptides or small molecules in nanoparticles, targeted modulation of DC phenotype and function has shown potential in preclinical models to achieve sustained immunoregulatory effects without generalized immunosuppression.^30^ Nanoparticles, including PLGA-based nanoparticles, have been widely used for tolerization to prevent autoimmunity. Here, we leverage past work and extend our previous work^30^ to show that Agg-CLNP enhance the effect of preventing RA onset and modulating flares. Our approach does not require cell manufacturing, avoids generalized immunosuppression, and could be formulated as an off-the-shelf nanoparticle formulation. Agg-CLNP have favorable features in terms of the cost of manufacturing and potential toxicity.

For successful translation, sterility and stability are key considerations for a potential therapeutic.^48^ To this end, Agg-CLNP were sterile filtered and stored prior to administration. Agg-CLNP were also stable at −20 °C, as assessed by DLS and UHPLC for at least 28 days post synthesis, supporting the potential of an off-the-shelf formulation. As several of the individual components have been well-studied, regulatory studies during future development stages could potentially leverage safety data of the extensively utilized components that are in the public domain and regulatory precedent for gaining approval. The active ingredient, calcitriol, is therapeutically used as a topical ointment for psoriasis.^49^ However, calcitriol’s serum half-life of 3–6 h makes its use for RA challenging.^50^ Consistent with prior reports, here, calcitriol encapsulated in Agg-CLNP is more readily delivered to the joint-proximal lymph nodes.^51,52^ The treatment was localized as Agg-CLNP treatment did not affect the endogenous systemic calcitriol concentration.

Agg-CLNP strongly modulated mouse BMDC and healthy human and RA patient MDC by reducing costimulatory molecules (CD40, CD80 and CD86), and major histocompatibility complex 2 (MHC2 in mice and HLA2 in humans), and increasing CTLA-4. While calcitriol and Agg-CLNP were dose matched to 5 nM calcitriol, there were differences in immunomodulation. The observed differences are likely due to the differences in bioavailability of calcitriol. Free calcitriol (solubilized in DMSO) is fully bioavailable upon addition to DC culture. On the other hand, calcitriol encapsulated in Agg-CLNP would either release from Agg-CLNP and be taken up by DC or released from Agg-CLNP after uptake by DC. It is likely that not all Agg-CLNP added to culture are taken up by DC and the poor solubility of calcitriol reduces the efficiency of uptake by DC. Thus, even though free calcitriol and Agg-CLNP are dose matched, differences in bioavailability likely contribute to the observed differences.

To maximize the accumulation of Agg-CLNP in the joint draining lymph nodes while minimizing systemic exposure, we selected a local route of administration. Intramuscular injections are a commonly used route of administration. In mice, i.m. injections allow for injecting more volume (up to 50 μL) compared to intra-articular injections. The i.m. injections were administered in the biceps femoris, which is in close proximity to the ankle-draining popliteal and inguinal lymph nodes and leads to CLNP accumulation, as we have demonstrated previously.^30^ While it is possible that subcutaneous injections could also achieve joint proximal lymph node delivery, we did not assess this route of administration due to the thinner mouse dermis and higher compliance of mouse skin compared to humans, which is known to lead to faster systemic absorption.^53^

Preventing progression from pre-RA to RA using immunosuppressants is an area of focus but risk/benefit considerations can hamper clinical adoption even when primary outcomes are achieved. For example, in a clinical trial that recruited subjects with PRObable RA, patients received either a first-line standard of care DMARD Methotrexate or Placebo Treatment (PROMPT).^54^ While onset of RA in subjects treated with methotrexate was significantly delayed, the difference in complete remission was small and raised concerns for overtreatment with methotrexate. In the “Prevention of clinically manifest rheumatoid arthritis by B-cell directed therapy in the earliest phase of the disease” (PRAIRI) study, a single dose of rituximab in individuals with pre-RA was found to significantly delay arthritis onset by up to 12 months, but this effect was transient.^55^ We investigated the enhancement of CTLA-4 IgG (clinically known as abatacept) with Agg-CLNP in preventing RA onset and severity. Combination therapies are an emerging strategy in RA treatment, aiming to enhance efficacy while minimizing adverse effects.^56^ As CTLA-4 was routinely shown to be upregulated by both flow cytometry and RNA-seq in DC, we anticipated that Agg-CLNP would synergize with CTLA-4 IgG therapy. SKG mice are well poised to model high risk patients, as these mice are genetically predisposed to develop RA post mannan injection and older SKG mice have been shown to naturally develop RA.^57,58^ Therefore, we injected SKG mice with CTLA-4 IgG or a combination of CTLA-4 IgG with Agg-CLNP at the time of RA synchronization and for two more days thereafter. While CTLA-4 IgG alone did significantly reduce the clinical score and ankle thickness, only the combination of CTLA-4 IgG with Agg-CLNP significantly reduced the disease onset and effectively prevented mice from attaining a high disease score (clinical score >1). These enhanced effects were also shown in the histopathology, where only the combination of CTLA-4 IgG and Agg-CLNP reduced synovitis, bone erosion, and proteoglycan loss in the hind paws. Agg-CLNP’s ability to synergize with CTLA-4 IgG in preventing RA severity and onset suggests potential for combination therapies. Given the established clinical use of CTLA-4 IgG in pre-RA, the addition of Agg-CLNP could enhance therapeutic outcomes by targeting complementary pathways.^59^ This approach may also allow for dose reduction of abatacept, potentially improving safety profiles and reducing treatment costs.

In contrast to RA flares, most rodent RA models rely on defined antigens.^60^ Arthritic SKG mice are a better mimic of RA as the pathology is not associated with a specific autoantigen but rather is associated with an inflammatory stimulus, making it more suitable for investigating human RA.^57,58^ SKG arthritis can be accelerated and synchronized by i.p. injection of mannan, a yeast-derived polysaccharide which stimulates DC and induces their maturation for subsequent T cell activation.^61–63^ Therefore, we developed the dexamethasone withdrawal flare model in SKG mice. Strikingly, we observed that repeated dexamethasone injections lose effectiveness in controlling arthritis in SKG mice suggesting that steroid tolerance might occur. While Agg-CLNP alone proved ineffective at mitigating arthritis with a clinical score >1, it was effective as a preventive, and we sought to assess whether it enhanced durability of the inflammation-resolving effects of dexamethasone in SKG mice. Agg-CLNP, when administered at the peak effectiveness of dexamethasone, mitigated the dexamethasone withdrawal flare and, importantly, retained its effectiveness over time. The immunophenotype associated with these effects were both immunomodulatory DC in the draining lymph nodes as well as lowered T_H_17 cells in the hind paws. The reduction of T_H_17 cells was localized to the site of disease, and not systemically in other tissues. While coadministration of dexamethasone and Agg-CLNP modulated arthritis severity, it did not modulate the phenotype of lymph node DC in the same manner as administration of Agg-CLNP post dexamethasone. This study supports the hypothesis that a low disease state, achieved here with dexamethasone, is necessary before administration of Agg-CLNP for durable clinical efficacy. Cy5-CLNP administration into SKG mice and into SKG mice following dexamethasone showed that dex did not affect the DC ability to uptake the particles and showed that the removal of the Agg peptide from the formulation obliterated the flare protective effects. Together, these results demonstrate that Agg-CLNP synergizes with dexamethasone to prevent a dexamethasone withdrawal flare, without systemic immune suppression. This addresses an unmet need in the clinic, where corticosteroids are used to suppress flares, but do not prevent flare recurrence.

Next generation bulk RNA-seq of the dendritic cells isolated from the draining lymph nodes post dexamethasone and post a combination of dexamethasone followed by Agg-CLNP, revealed potential genes associated with the flare protective effects of Agg-CLNP. The upregulated genes in mice treated with Agg-CLNP included *Ctla4*, *Ccl22*, *Cd200*, and *Tsc22d3*. The downregulated gene of interest in mice treated with Agg-CLNP was *Card14*. We have observed CTLA-4 increases on DC treated with Agg-CLNP in human samples and murine samples. While CTLA-4 is classically described on T cells, CTLA-4 on DC has been reported to result in regulatory functions through altered antigen presentation and modulated cell function.^64,65^ *Ccl22* encodes a chemokine that acts as a chemoattractant for T_regs_, T_H_2, and monocytes.^66^ This gene is upregulated in tolerogenic DC and can suppress inflammation by the attraction of immunosuppressive T_regs_.^67^ *Cd200* in DC encodes a membrane glycoprotein that interacts with the CD200 receptor (CD200R) on macrophages and other myeloid cells to inhibit immune activation and induce T_regs_.^68^ High CD200 expression is associated with tolerogenic DC, and loss of CD200 signaling has been associated with rheumatoid arthritis.^69,70^ *Tsc22d3* encodes glucocorticoid-induced leucine zipper (GILZ), a key glucocorticoid-induced transcription factor involved in anti-inflammatory and immunosuppressive pathways. GILZ suppresses nuclear factor kappa B (NF-κB) and activator protein 1 (AP-1) signaling, reducing DC activation and pro-inflammatory cytokine production.^71^ Notably, suppression of NF-κB is the mechanism of calcitriol’s anti-inflammatory effects as well.^33,34^ As *Tsc22d3* remained upregulated in DC treated with Agg-CLNP post dexamethasone, it suggests that Agg-CLNP may be maintaining the anti-inflammatory phenotype imposed by dexamethasone. *Card14* encodes caspase recruitment domain family member 14 (CARD14), a scaffolding protein involved in NF-κB signaling, mainly expressed in keratinocytes.^72^ Gain-of-function mutations in CARD14 have been associated with psoriasis and other inflammatory skin diseases, suggesting a role in DC-mediated inflammation.^73^ As CARD14 acts as an immune amplifier in inflammatory conditions, and enhances NF-κB activation, its downregulation in Agg-CLNP treated DC likely contributes to the clinical effects observed in the dexamethasone withdrawal flare model. Identifying these targets could further inform patient stratification and optimize therapeutic strategies, enhancing Agg-CLNP’s clinical relevance.

## Methods

### Study Design

The objective of this study was to develop an immunomodulatory agent to modulate arthritis flares in a dexamethasone flare withdrawal model of rheumatoid arthritis and to reduce arthritis onset and severity in combination with human CTLA-4 IgG. We also sought to show the efficacy of the immunomodulatory agent on both healthy and patient human dendritic cells. To this end, we formulated Agg-CLNP. All cell culture studies were performed with a minimum of three technical replicates. For *in vivo* studies, outcomes were determined by assessing clinical scores and ankle thickness. For SKG arthritis studies, littermate mice were injected with mannan to synchronize disease onset. The criteria for omission were (i) signs of arthritis on day 0 and (ii) failure to develop arthritis by the first treatment time point. All other animals were included in the data analysis. End points for data collection were based on changes in and progression of clinical scores in the treatment groups. Sample size for each individual experiment is provided in the figure legends. To achieve adequate power, all mouse arthritis studies were conducted by combining at least two age-matched litters. In general, statistical power for arthritis studies was based on prior reports of and our experience with arthritis mouse models. No unexpected or unusual safety hazards were encountered.

### Materials

Poly­(lactide-*co*-glycolide)-polyethylene glycol-maleimide (50:50 L:G 20kD PLGA, 2kD PEG SKU: 2794-20K-2000-1g, lot: 2794200204) was purchased from NanoSoft polymers. Calcitriol (71820, lot: 0601887-55) was purchased from Cayman Chemical. Cy5-polyethylene glycol-thiol (2kD, FL078003-2K, lot: 20201217BL05,) was purchased from Biochempeg. Dimethyl sulfoxide (DMSO, D128-500, lot:194474) and acetonitrile (A998-1, lot: 206498) were purchased from Fischer Chemical. Mannan (M7504-5G, lot: 0000401484), lipopolysaccharide (LPS, L3012-5MG, lot: 0000091258), and fetal bovine serum (FBS, F2442-500 ML, lot: 21G126) were purchased from Sigma-Aldrich. RPMI powder (31800-022, lot: 2917359), beta mercaptoethanol (21985-023, lot: 2603102), MEM NEAA (11140-050, lot: 2696374), and sodium pyruvate (11360-070, lot: 2813888) were purchased from Gibco. Dialysis sacs (12kD, D6191-25EA) and collagenase (C2139-1G, lot: 0000367377) were purchased from Millipore Sigma. 6-well culture plates (353046) and 96-well culture plates (FB012931) were purchased from Fisher. GM-CSF (315-03-250UG, lot: 032255) was purchased from Peprotech. Dexamethasone (501012) was purchased from VetOne. Human CTLA-4 IgG (BE0099, lot: 826622J1) and human IgG (BE0096, lot: 829223N1) were purchased from BioXCell. Ficoll gradient separation buffer (25-072-CV, lot: 30324002) was purchased from Corning. Calcitriol ELISA kit (NBP2-82432) was purchased from Novus Biologicals. TriZol LS (10296010, lot: 96739103) was purchased from Ambion. Human monocyte isolation kit (19359, lot: 1000172465) and Easy Sep buffer (20144, lot: 1000193044) were purchased from StemCell. The 70 μm cell strainers (881-10010-PK, lot: c4070) were purchased from MTCBio. Fixation concentrate (00-5123-43, lot: 2766743), fixation diluent (00-522-56, lot: 2831093), and permeabilization buffer (00-8333-56, lot: 2911220) were purchased from Invitrogen. DNase I (10104159001, lot: 80781100) was purchased from Roche. EDTA coated microtubes (365974, lot: 2181885) were purchased from BD. Buffy coats were obtained from the Stanford Blood Bank.

### Mouse Models

All animal work was approved by the UCSD Institutional Animal Care and Use Committee (IACUC) under protocol #S17160 and followed the National Institutes of Health guidelines and relevant AALAC-approved procedures. BALB/c SKG mice were obtained through a Materials Transfer Agreement between UC San Diego and Kyoto University and colonies were maintained at UCSD. BALB/c SKG mice used were both male and female. In each study, mice used were either all males or all females.

Arthritis in SKG mice was synchronized in 8–12-week-old SKG mice via intraperitoneal (i.p.) injection of 20 mg mannan dissolved in 200 μL of sterile PBS. Disease severity was determined twice weekly using clinical scoring and measurement of hind paw swelling using calipers while mice were anesthetized. Fore and hind paws were assessed independently in each mouse and were assigned scores according to the following criteria: no visible swelling (0), mild to moderate swelling (0.5), severely swollen (1.0), as well as an additional for 0.1 for each swollen digit. Clinical scores reported are the aggregate of all paws (maximum of 5.8) from a single mouse unless otherwise noted. A score of 5.2 was considered the clinical end point and mice who attained this score before the end of the study were sacrificed according to IACUC guidelines.

### Human Patient Samples

Rheumatoid arthritis patient samples were taken at the Cedars-Sinai Medical Center with informed consent under an IRB-approved protocol (PI: Dr. Jon T. Giles).

### Aggrecan Calcitriol Loaded Nanoparticle (Agg-CLNP) Synthesis

Cysteine-terminated aggrecan peptide (Peptide 2.0) was added in a 1:1 molar ratio to 20 mg of PLGA–PEG-MAL and dissolved in 1 mL of DMSO. This mixture was agitated overnight. 60 μL of 1 mg/mL calcitriol in DMSO was then added to the polymer solution. The polymer solution was then diluted with 2 mL of DMSO and 3 mL of ethanol. The polymer solution was then added dropwise via syringe pump (SyringePump.com, Model 4000) to 40 mL of homogenizing Milli-Q water at 3500 rpm (Silverson, L5M-A) and allowed to come to homogeneity for 10 s. The nanoparticle solution was then transferred to a 12 kDa dialysis bag and placed in a 6 L water bath. The water bath solution was changed every 3 h for a total 9-h dialysis against 18 L of water. Sucrose was added to bring the total weight percent of sucrose to 10%. The Agg-CLNP solution was then sterile filtered, aliquoted, and stored at −20 °C. Thawed aliquots were used for each study.

To formulate Cy5-CLNP, the above procedure was followed but with Cy5-PEG-thiol rather than cystine-terminated aggrecan peptide.

### Agg-CLNP Characterization

An aliquot of undiluted nanoparticles was added to a cuvette and placed in a Malvern Zetasizer Pro for dynamic light scattering analysis. Measurements with the Zetasizer Pro utilize ZS XPLORER software. Encapsulation of calcitriol in Agg-CLNP was determined on an Thermo Vanquish UHPLC (ThermoFischer Scientific). Briefly, thawed nanoparticle suspensions without sucrose were spun down at 21100 g for 10 min in a centrifuge. The supernatant was aspirated, and the pellet dissolved in HPLC grade acetonitrile. The solutions were run in a Ascentis Express 90Å C18 reverse phase column (MilliporeSigma, Cat#53825-U, lot: USWM003951) with a mobile phase of 100% acetonitrile at an isocratic flow rate of 0.1 mL/min at a detection wavelength of 265 nm. Area under the curve values were compared to a standard plot of known calcitriol concentrations run in the same conditions. The encapsulation efficiency (*ee*) of calcitriol in the formulations was calculated using the following equation:

$$ee\;\left(\%\right)=\frac{Calcitriol\;concentration\;measured\;in\;C\mathrm{LN}P}{Calcitriol\;concentration\;in\;reaction}\times 100$$

### Calcitriol Plasma Concentration Post Agg-CLNP Administration

50 μL of Agg-CLNP solution (equivalent to 22 μg of Agg-CLNP) was injected intramuscularly into each biceps femoris of 6 SKG mice (for a total of 44 μg in each mouse). At both 1- and 24 h post injection, a terminal retroorbital blood collection was performed with a heparinized capillary into EDTA-coated blood collection tubes. Blood was also collected from three SKG mice that did not receive Agg-CLNP injections to determine endogenous calcitriol concentration. Blood samples were spun at 1000 g for 10 min to separate the blood cells from the plasma. Plasma samples were analyzed for calcitriol concentration with a calcitriol ELISA detection assay from Novus Biologicals (NBP2–82432) following the manufacturer’s instructions.

### *In Vitro* Mouse Dendritic Cell Differentiation Assay

SKG mouse bone marrow cells were harvested by homogenizing the long bones using a mortar and pestle in complete 1640 RPMI media with 10% FBS and 20 ng/mL GM-CSF. The homogenate was strained through a 70 μm cell strainer. The strained solution was diluted to 2,000,000 cells/mL with media and 2 mL were added per well to a tissue culture treated 6-well plate. In a subset of wells, Agg-CLNP was added to achieve a 1 nM calcitriol concentration in the culture. Plates were incubated at 37 °C at 5% CO_2_. On day 3 of the culture, the wells were supplemented with 2 mL of fresh complete 1640 RPMI media containing GM-CSF and fresh Agg-CLNP to maintain experimental concentration. On day 6 of the culture, half of the media was carefully removed and 2 mL of fresh media and fresh Agg-CLNP were added to maintain experimental concentration. LPS was added at 50 ng/mL LPS for BMDC activation. After overnight activation, BMDC were analyzed by flow cytometry.

### *In Vitro* Human Dendritic Cell Differentiation Assay

Human Buffy coats or whole blood from RA patients were obtained and peripheral blood mononuclear cells were isolated by gradient centrifugation with Ficoll separation buffer (Corning). Briefly, buffy coat or whole blood was diluted 1:1 with 2% FBS in 1xPBS. Fifteen mL of Ficoll buffer was added to a 50 mL conical vial and 30 mL of diluted human sample was carefully layered on top of the Ficoll. The gradient separation was performed at 800 g at room temperature for 20 min with the brake off. The peripheral blood mononuclear cells were carefully removed from the Ficoll layer and washed with 2% FBS in 1xPBS at 300 g for 8 min at room temperature. A second wash with 2% FBS in 1xPBS was performed at 120 g for 10 min at room temperature with the brake off to remove platelets. A human monocyte isolation kit (StemCell, PN: 19359, Lot: 1000172465) was utilized to enrich CD14^+^ monocytes. Monocytes were suspended at 1,000,000 cells/mL in complete 1640 RPMI with 10% FBS, 1x MEM NEAA, 1x sodium pyruvate and 20 ng/mL murine GM-CSF. 100 μL of the monocyte solution was added per well to a 96-well tissue culture treated flat bottom plate and incubated at 37 °C in 5% CO_2_. On day 3, the supernatant was removed, and fresh media was added. On day 4, calcitriol (5 nM), dexamethasone (1 μM), or Agg-CLNP (dose matched to 5 nM calcitriol) was added. On day 5, LPS was added to each well to achieve 500 ng/mL LPS, Agg-CLNP (dose matched to 5 nM calcitriol) was added to the dex+Agg-CLNP wells at this time. On day 7, the activated MDC were analyzed by flow cytometry.

### Prophylactic versus Treatment Timelines of Agg-CLNP in SKG Arthritis

To assess the effect of Agg-CLNP with either a prophylactic or treatment timeline in the SKG model of autoimmune arthritis, littermate 8–12-week-old female SKG mice were injected i.p. with 20 mg of mannan on day 0 to synchronize arthritis induction. For prophylactic studies, a subset of SKG mice was injected i.m. with Agg-CLNP (22 μg/day) into each biceps femoris once a day for 3 days prior to mannan injection on day 0. These mice were clinically scored twice a week for 2 weeks prior to sacrifice. For treatment studies, a subset of SKG mice was injected i.m. with Agg-CLNP into each biceps femoris (22 μg/day) on days 14–16 post mannan injection on day 0. These mice were clinically scored twice a week for 26 days post mannan injection.

### Arthritis Prevention with Agg-CLNP in Combination with CTLA-4 IgG

To assess the effectiveness of Agg-CLNP in combination with CTLA-4 IgG in SKG arthritis onset and severity, littermate 8–12-week-old female SKG mice were injected i.p. with 20 mg of mannan on day 0 to synchronize arthritis induction. On days 0–2 a subset of mice was injected i.p. with human IgG as a control (250 μg/day). On days 0–2 a subset of mice was injected i.p. with human CTLA-4 IgG (250 μg/day). On days 0–2 a subset of mice treated with the CTLA-4 IgG was injected i.m. with Agg-CLNP into each biceps femoris (22 μg/day). Clinical scores were assessed for 14 days post mannan injection. On day 14, mice were sacrificed, and the inguinal and popliteal lymph nodes were extracted for flow cytometry analysis and the ankles were fixed in 4% paraformaldehyde.

### Dendritic Cell Profile in Lymph Nodes of SKG Mice

To assess the dendritic cell profile in lymph nodes post dexamethasone and post dexamethasone followed by Agg-CLNP in SKG arthritis, littermate 8–12-week-old female SKG mice were injected i.p. with 20 mg of mannan on day 0 to synchronize arthritis induction. On days 8–10 a subset of mice was injected i.p. with dexamethasone (125 μg/day). On days 11–13 a subset of dexamethasone treated mice were injected i.m. with Agg-CLNP into each biceps femoris (22 μg/day). Clinical scores were assessed twice a week for 14 days post mannan injection. On days 11 and 14 a subset of mice was sacrificed, and popliteal and inguinal lymph nodes were harvested. Lymph nodes from two mice of the same treatment group were combined for flow sample preparation.

To assess the dendritic cell profile in lymph nodes, littermate 8–12-week-old female SKG mice were injected i.p. with 20 mg of mannan on day 0 to synchronize arthritis induction. On days 8–10 a subset of mice was injected i.p. with dexamethasone (125 μg/day) and a subset of dexamethasone treated mice were injected i.m. with Agg-CLNP into each biceps femoris (22 μg/day). Clinical scores were assessed twice a week for 14 days post mannan injection. On day 14, mice were sacrificed, and popliteal and inguinal lymph nodes were harvested. Lymph nodes from two mice of the same treatment group were combined for flow sample preparation.

To assess Cy5-CLNP uptake in lymph node DC, littermate 8–12-week-old female SKG mice were injected i.p. with 20 mg of mannan on day 0 to synchronize arthritis induction. On days 8–10 a subset of mice was injected i.p. with dexamethasone (125 μg/day). On days 11–13, all mice were injected i.m. with Cy5-CLNP into each biceps femoris (22 μg/day). Clinical scores were assessed twice a week for 14 days post mannan injection. On day 14, all mice were sacrificed, and popliteal and inguinal lymph nodes were harvested.

### Multiple Flare Treatment with Agg-CLNP Post Dexamethasone

To assess the efficacy of Agg-CLNP in SKG arthritis flare prevention post dexamethasone injection, littermate 8–12-week-old female SKG mice were injected i.p. with 20 mg of mannan on day 0 to synchronize arthritis induction. On days 8–10, 22–24, and 36–38 a subset of mice was injected i.p. with dexamethasone (25 μg/day). On days 11–13, 25–27, and 39–41 a subset of dexamethasone treated mice were injected i.m. with Agg-CLNP into each biceps femoris (22 μg/day). Clinical scores and ankle thickness were assessed twice a week for 42 days post mannan injection. On day 42, mice were sacrificed, and ankles were fixed in 4% paraformaldehyde. Mice from the dexamethasone+Agg-CLNP combination group that were no longer responsive to dexamethasone injections (change in clinical score ≤ 0.5) were excluded from the histology analysis.

### Flare Treatment with Agg-CLNP in Combination with Dexamethasone for TH17 Assessment

To assess the effect of Agg-CLNP on T_H_17 counts in SKG post dexamethasone remission, littermate 8–12-week-old female SKG mice were injected i.p. with 20 mg of mannan on day 0 to synchronize arthritis induction. On days 8–10, all mice were injected i.p. with dexamethasone (125 μg/day). On days 11–13 a subset of dexamethasone treated mice were injected i.m. with Agg-CLNP into each biceps femoris (22 μg/day). Clinical scores and ankle thickness were assessed twice a week for 14 days post mannan injection. On day 14, mice were sacrificed and spleens, inguinal lymph nodes, popliteal lymph nodes, forepaws, and hind paws were harvested for flow cytometry.

### Bulk RNA-seq and Analysis of DC from Lymph Nodes of SKG Mice

To assess the dendritic cell gene profile in lymph nodes post dexamethasone and post a combination of dexamethasone and Agg-CLNP in SKG arthritis, littermate 8–12-week-old female SKG mice were injected i.p. with 20 mg of mannan on day 0 to synchronize arthritis induction. On days 8–10 a subset of mice was injected i.p. with dexamethasone (125 μg/day). On days 11–13 a subset of dexamethasone treated mice were injected i.m. with Agg-CLNP into each biceps femoris (22 μg/day). Clinical scores were assessed twice a week for 14 days post mannan injection. On day 14, the mice were sacrificed, and popliteal and inguinal lymph nodes were harvested. Lymph nodes from four mice of the same treatment group were homogenized for staining and cell sorting. Cell suspensions were stained with Zombie Aqua for 10 min in 1xPBS and CD11c on AlexaFlour594 for 30 min in FACS buffer. Zombie Aqua^–^CD11c^+^ cells were sorted with a FACSAriaII at the La Jolla Institute for Immunology into fetal bovine serum. Sorted cells were spun down at 400 g for 5 min and resuspended in 500 μL of TriZol LS before freezing.

Frozen cells in TriZol LS were shipped to the Cedars-Sinai Applied Genomics, Computation and Translational Core for sequencing. For standard input mRNA-Seq, RNA was isolated and normalized to 460 ng before undergoing poly-A selection using NEBNext Magnetic Oligo d­(T)­25 Beads. Poly-A selected mRNA was prepared for sequencing using the IDT xGen Broad Range RNA Library Prep Kit with the IDT Normalase Unique Dual Indexing Primer Plate and 11 cycles of PCR amplification. For Low Input mRNA-Seq, RNA samples were normalized to ∼ 250 pg and prepared for sequencing using the Takara Smart-Seq mRNA LP (with UMIs) kit. cDNA was PCR amplified for 13 cycles followed by Indexing PCR for 15 cycles using the Takara Unique Dual Index Kit. The sequencing was performed on an Illumina NovaSeq X.

Analysis of the raw reads from RNA-seq prepared libraries was done at UCSD using the nf-core/rnaseq pipeline (v3.17.0). FASTQ files were aligned to the mm10 reference genome using STAR, and transcript quantification was performed using Salmon. Adapter and quality trimming were conducted using Trim Galore. Duplicate reads were removed using MarkDuplicates from Picard tools. Count normalization and differential RNA-seq analysis were performed using DESeq2 after removing nonexpressed and lowly expressed genes. Differentially expressed genes were filtered based on an adjusted p-value (≤0.05) and a log_2_ fold-change (≥1.25).

### Histological Processing

After sacrifice, mouse hind limbs were excised below the knee joint. Muscle and skin were removed to the highest degree possible without damaging internal structures, and the limbs were fixed in 4% paraformaldehyde (PFA) for 24 h. The fixed limbs were then transferred to a 70% ethanol solution. Samples were then sent to the University of Gothenburg where they were decalcified and embedded in paraffin. Paraffin embedded limbs were sectioned to an appropriate depth according to SMASH guidelines and stained with hematoxylin and eosin or toluidine blue using standard tissue processing techniques. Stained slides were digitized using a ZEISS Axioscan 7 Digital Whole Slide Scanner.

### Histomorphometry Analysis

For synovitis, proteoglycan loss scoring and bone erosion scoring, SMASH guidelines were followed. Briefly, histological sections were examined and proteoglycan loss was scored as follows: 0 – healthy intact cartilage consisting of fully stained cartilage layer with a smooth surface; 1 – Mild loss of staining in ∼ 1/3 of the superficial cartilage zone, still predominantly blue with toluidine blue; 2 – Moderate loss of toluidine blue staining in up to 2/3 of the superficial cartilage zone; 3 – Complete loss of toluidine blue staining in the superficial cartilage zone. Bone erosion was scored as follows: 0 – Healthy, intact bone surface; 1 – Small, superficial bone erosion at the outer surface of the bone, no breakage into marrow; 2 – Enhanced local bone erosions into subchondral space, partial or complete penetration of cortical bone; 3 – Massive enlarged subchondral bone erosion, extended synovial pannus invasion causing near-complete breakthrough of cortical bone to the marrow. Synovitis was scored as follows: 0 - healthy, one to two cell layers of synovial membrane, no inflammatory infiltrates; 1 - three to five cell-layered synovial membrane, mild cellular infiltrate into the synovium and exudate in the joint cavity with low cell density; 2 - multilayered synovial membranes, enhanced cellular infiltrates and increased cell density throughout the joints; 3 - severely expanded inflammation filling all joint cavities, hyperplastic synovial tissue with high cell density; 4 - maximally expanded inflammation filling all joint cavities, hyperplastic synovial tissue with high cell density. Scoring was performed by a treatment-blinded operator.

### Flow Cytometry Analysis

Antimouse antibodies against CD4 (PN: 100428, clone: GK1.5, lot: B347337), CD45 (PN: 103130, clone: 30-F11, lot: B349380), CD11b (PN: 101235, clone: M1/70, lot: B360998), CD11c (PN: 117346, clone: N418, lot: B325181), CD80 (PN: 104705, clone: 16-10A1, lot: B334893), CD86 (PN: 105115, clone: GL-1, lot: B315643), I-A/I-E (MHC2) (PN: 107628, clone: M5/114.15.2, lot: B350373), CTLA-4 (PN: 106309, clone: UC10-4B9, lot: B357050), IL-17 (PN: 506916, clone: TCC11-18H10.1, lot: B358441), and ICOS (PN: 313519, clone: C398.4A, lot: B378504) were purchased from Biolegend. Antihuman antibodies against CD11c (PN: 337214, clone: Bu15, lot: B401287), CD40 (PN: 334320, clone: 5C3, lot: B383527), CD80 (PN: 305219, clone: 2D10, lot: B400367), HLA-2 (PN: 361715, clone: Tu39, lot: B423731), CTLA-4 (PN: 369633, clone: BNI3, lot: B352357), and CD86 (PN: 367607, clone: 590H11, lot: B399839) were purchased from Biolegend. All cells were gated based on forward and side scatter characteristics to limit debris, including dead cells. The Zombie Aqua Fixable Viability Kit (Biolegend, lot: B333785) stain was used to separate live and dead cells. Antibodies were diluted 1:400. Gates were drawn based on fluorescence-minus-one controls, and the frequencies of positively stained cells for each marker were recorded. Intracellular/intranuclear stains were performed by first staining for surface markers according to manufacturer’s protocols, then fixing and permeabilizing cells using a Fixation/Permeabilization Buffer Set (Invitrogen). To quantify immune cell subsets in mouse lymph nodes, lymph nodes were homogenized through a 70 μm cell strainer. To quantify immune cell subsets in mouse ankles, ankles were harvested after sacrificing mice, skin was removed, and ankles were incubated at 37 °C in a solution of complete RPMI, 1 mg/mL Type VIII collagenase and 0.1 mg/mL DNase I for 50 min with constant gentle agitation. The supernatant was filtered through a 70 μm cell strainer and subsequently stained for flow cytometry. To quantify immune cell subsets in the spleen, red blood cells (RBC) were first lysed with RBC lysis buffer before proceeding with staining. Flow cytometry was performed using an Attune NxT Acoustic Focusing cytometer analyzer (A24858) and data analyzed using FlowJo (BD) software.

### Statistics

Sample sizes for animal studies were based on prior work with SKG mice without the use of additional statistical estimations. Results were analyzed where indicated using one- or two- way ANOVA; paired Students *t*-test; unpaired Students *t*-test with Welsch’s correction; Mantel-Cox; and Kruskal–Wallis, each identified for each individual experiment in the figure legends. Data were analyzed using Graphpad Prism software.

## Supplementary Material



## Data Availability

All data are available in the main text or the Supporting Information. Sequencing data in this publication have been deposited in the NCBI’s Sequence Read Archive (SRA) database. RNA-Seq data are accessible through BioProject accession number PRJNA1249430.

## Notes

Flow gating strategy for murine BMDC, murine BMDC phenotype with Agg-CLNP, flow gating strategy for healthy human MDC, flow gating strategy for RA patient MDC, disaggregated RA patient MDC data, clinical scores of SKG given Agg-CLNP as prophylactic versus treatment, magnified regions of interest of representative histological scans of Figure 4, all histology scans used in Figure 4, flow gating strategy of CD4^+^ T cells from SKG tissues, ICOS^+^ on CD4^+^ T cells from lymph nodes of SKG, flow gating strategy for DC from SKG lymph nodes, immune cell counts from lymph nodes of SKG, co-administration of dexamethasone and Agg-CLNP data, Cy5-CLNP uptake in lymph node DC of arthritic SKG mice, T_H_17 counts from SKG tissues, magnified regions of interest of representative histological scans of Figure 6, all histology scans used in Figure 6, DLS of three Agg-CLNP batches, calcitriol *ee* of three Agg-CLNP batches, and RA patient donor characteristics (PDF)
