# Supplementary material for: Immunomodulatory Nanoparticles Enable Combination Therapies To Enhance Disease Prevention and Flare Control in Rheumatoid Arthritis
Source: ACS Cent Sci. 2025 Aug 6;11(9):1581–97. doi: 10.1021/acscentsci.5c00723 (PMC12464772; doi:10.1021/acscentsci.5c00723)
Supplement: Supplementary file 1 [file oc5c00723_si_001.pdf]

Supporting Information for Publication

Immunomodulatory nanoparticles enable combination therapies to enhance disease prevention and flare control in rheumatoid arthritis

Wade T. Johnson[1], Elizabeth L. Wilkinson[1], Neha Iyer[1], Maksim Dolmat[1], Miriam Bollmann[2], Nour Dada[2], Xiaofu Wei[3], Shen Yang[4], Tiffany Zhang[4], Grace Yoo[4], Marianne Bernardo[4], Madison Price[4], Elizabeth Frame[4], Mariko Ishimori[4], Jon T. Giles[4], Wei Wang[3,5], Mattias N.D. Svensson[2], Nunzio Bottini[4*] & Nisarg J. Shah[1*]

[1]Department of Chemical and Nano Engineering, University of California, San Diego, La Jolla, CA 92093, USA

[2]Department of Rheumatology and Inflammation Research, University of Gothenburg 41346, Sweden

[3]Department of Chemistry and Biochemistry, University of California, San Diego, La Jolla, CA

92093, USA

[4]Kao Autoimmunity Institute and Division of Rheumatology, Cedars-Sinai Medical Center, Los Angeles, CA 90048, USA

[5]Department of Cellular and Molecular Medicine, University of California, San Diego, La Jolla,

CA 92093, USA

*Corresponding authors: Nunzio Bottini (**nunzio.bottini@cshs.org**) and Nisarg J. Shah (**nshah@ucsd.edu**)

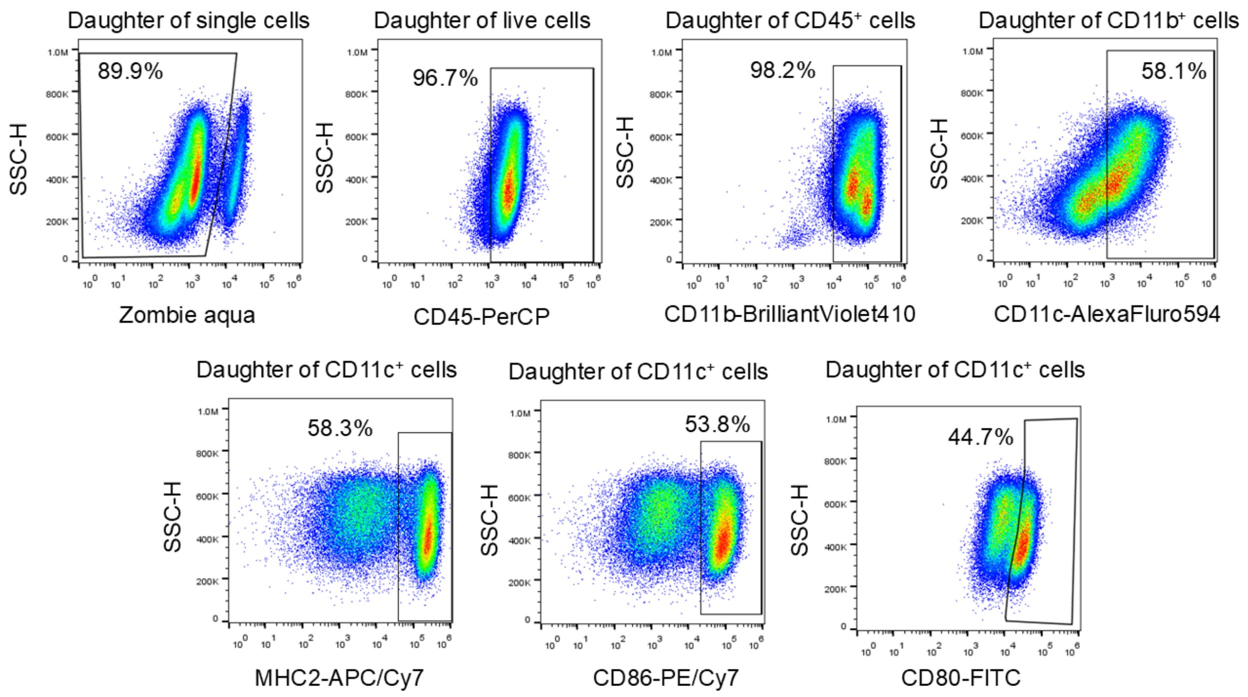

Figure S1. Flow gating strategy of murine BMDC.

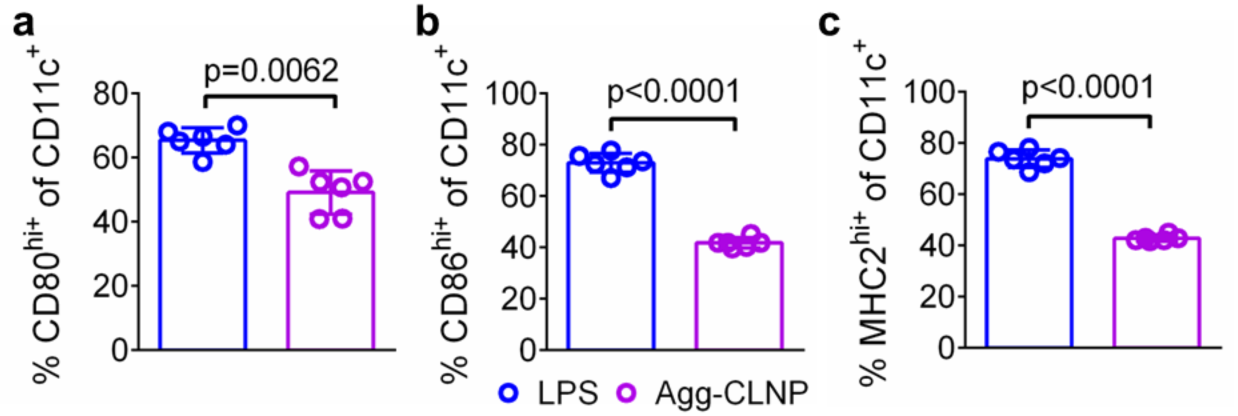

Figure S2. Agg-CLNP modulate DC from SKG *in vitro*. (a, b, and c) Costimulatory molecules (CD80 and CD86) and major histocompatibility complex II (MHC2) positivity on $CD11c^+$ DC after culture with LPS only or Agg-CLNP as measured by (a) CD80, (b) CD86, and (c) MHC2. Data are means±SD of six technical replicates. Statistical analyses were performed with paired Students *t*-test.

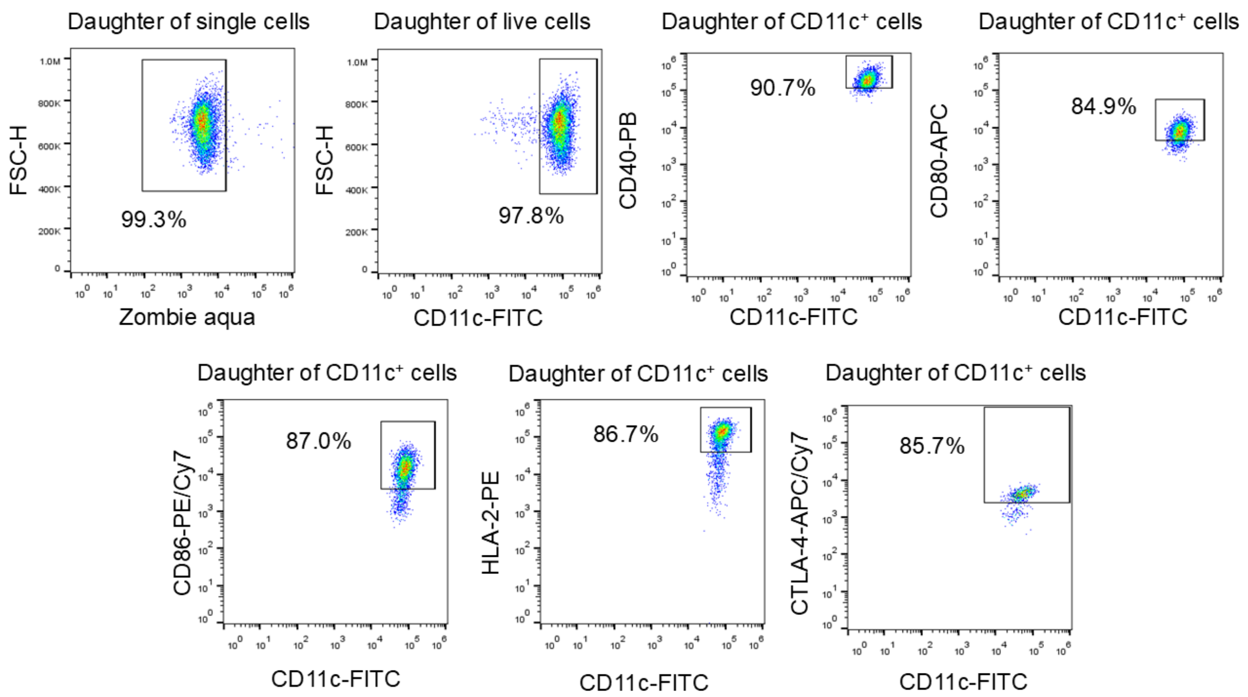

Figure S3. Flow gating strategy of healthy human MDC.

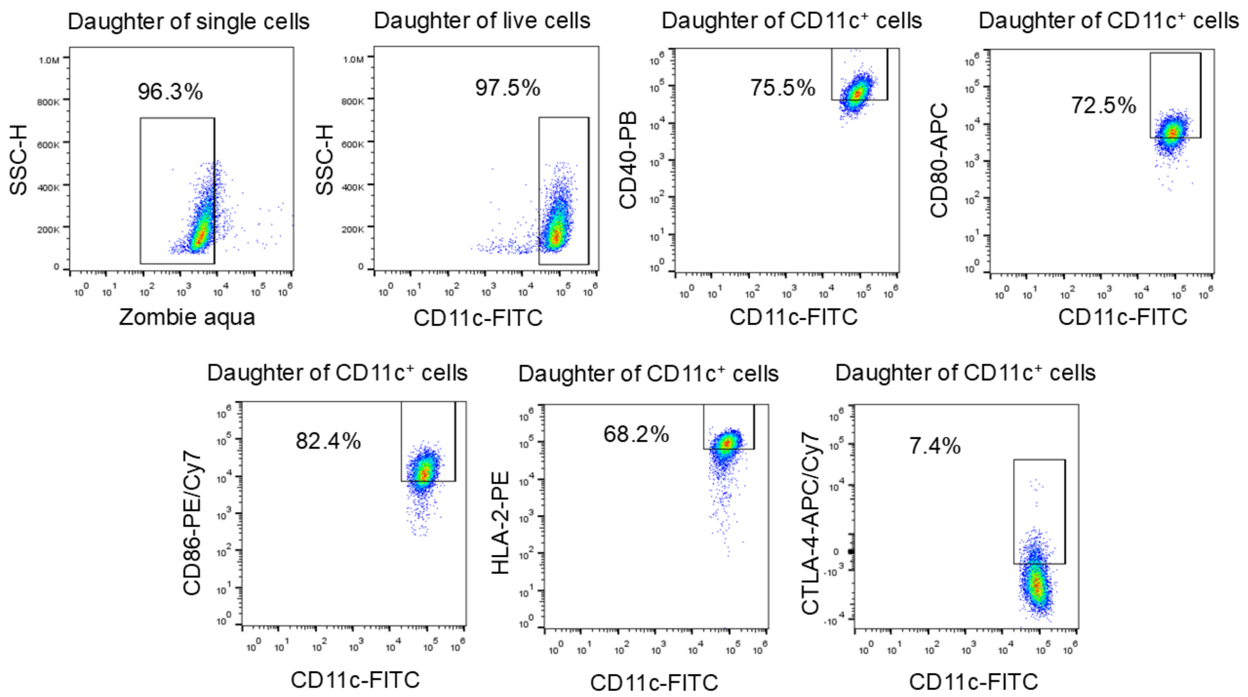

Figure S4. Flow gating strategy of RA patient human MDC.

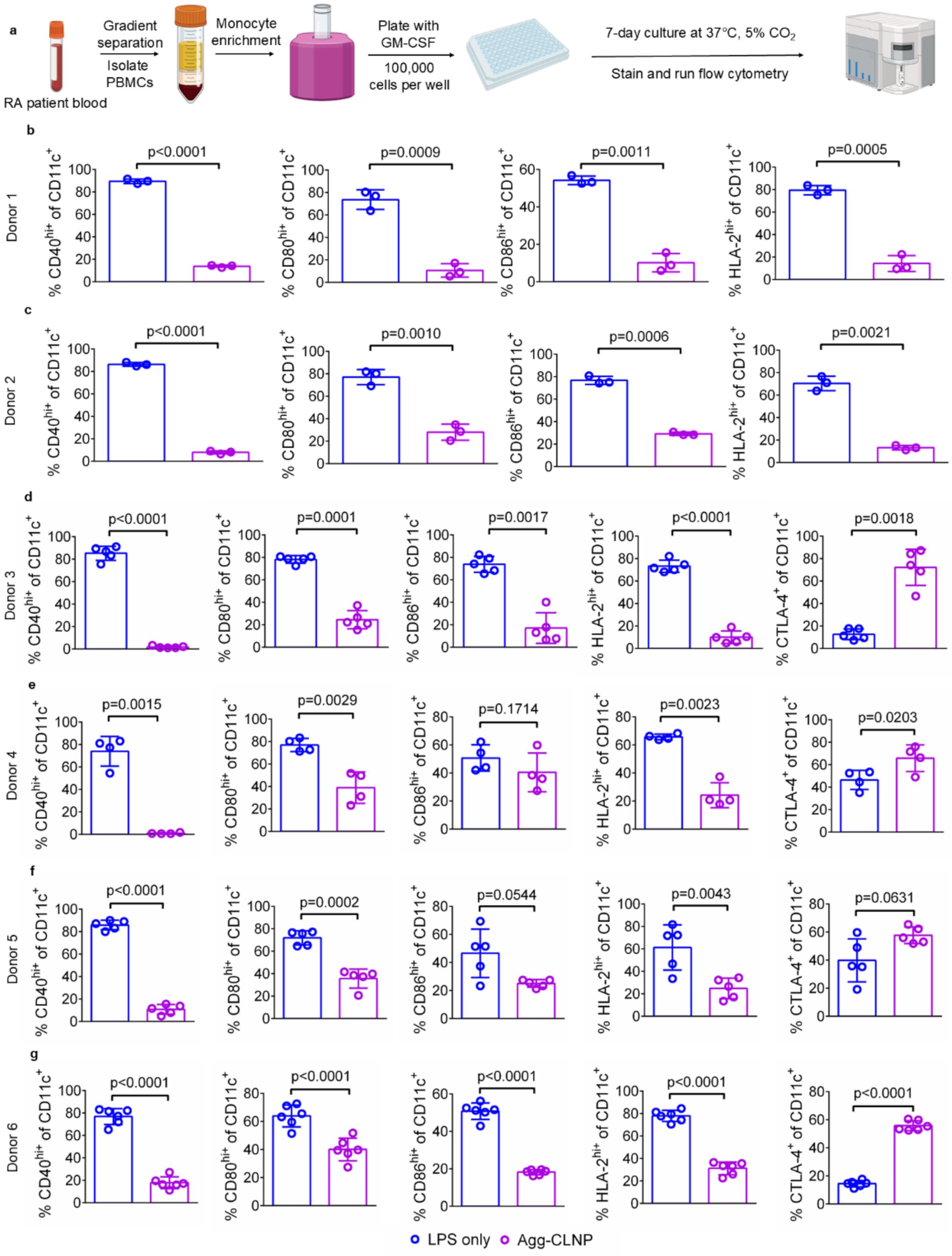

Figure S5. Agg-CLNP imparts a tolerogenic phenotype on RA patient human MDC *in vitro*, disaggregated. (a) Experimental schematic of MDC culture. (b, c, d, e, f and g) Costimulatory molecules (CD40, CD80 and CD86), human leukocyte antigen II (HLA-2), and cytotoxic T-lymphocyte-associated protein 4 (CTLA-4) positivity on CD11c$^+$ MDC after culture with LPS only or Agg-CLNP as measured by (b) donor 1, (c) donor 2, (d) donor 3, (e) donor 4, (f) donor 5, and (g) donor 6. Data are means±SD of (b-c) three technical replicates, (d & f) five technical replicates, (e) four technical replicates, and (g) six technical replicates from representative donors. Statistical analyses in (b-g) were performed with paired Students *t*-test. Schematic in (a) was composed using BioRender.

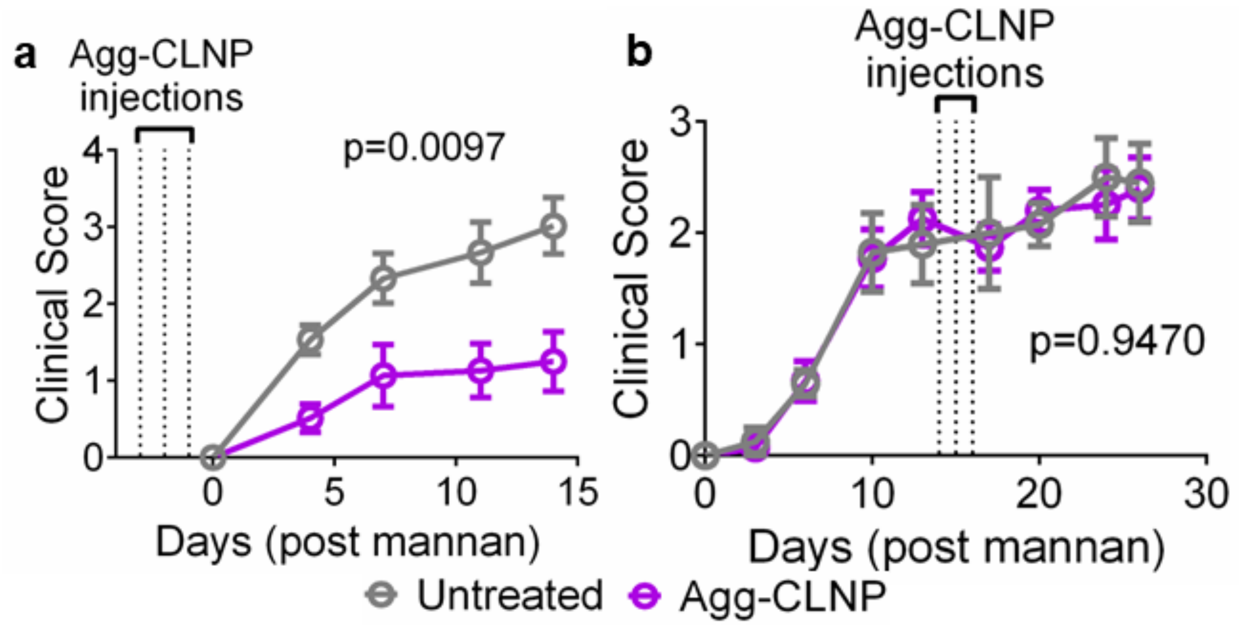

Figure S6. Agg-CLNP prophylactically modulate SKG arthritis. (a) Clinical scores of mice treated with Agg-CLNP prophylactically (22µg Agg-CLNP/biceps femoris/day, for three days prior to mannan, n=6) or untreated (n=6). (b) Clinical scores of mice treated with Agg-CLNP as a treatment (22µg Agg-CLNP/biceps femoris/day, days 14-16, n=7) or unteated (n=4). Data are means±SEM. Statistical analyses were performed using two-way ANOVA.

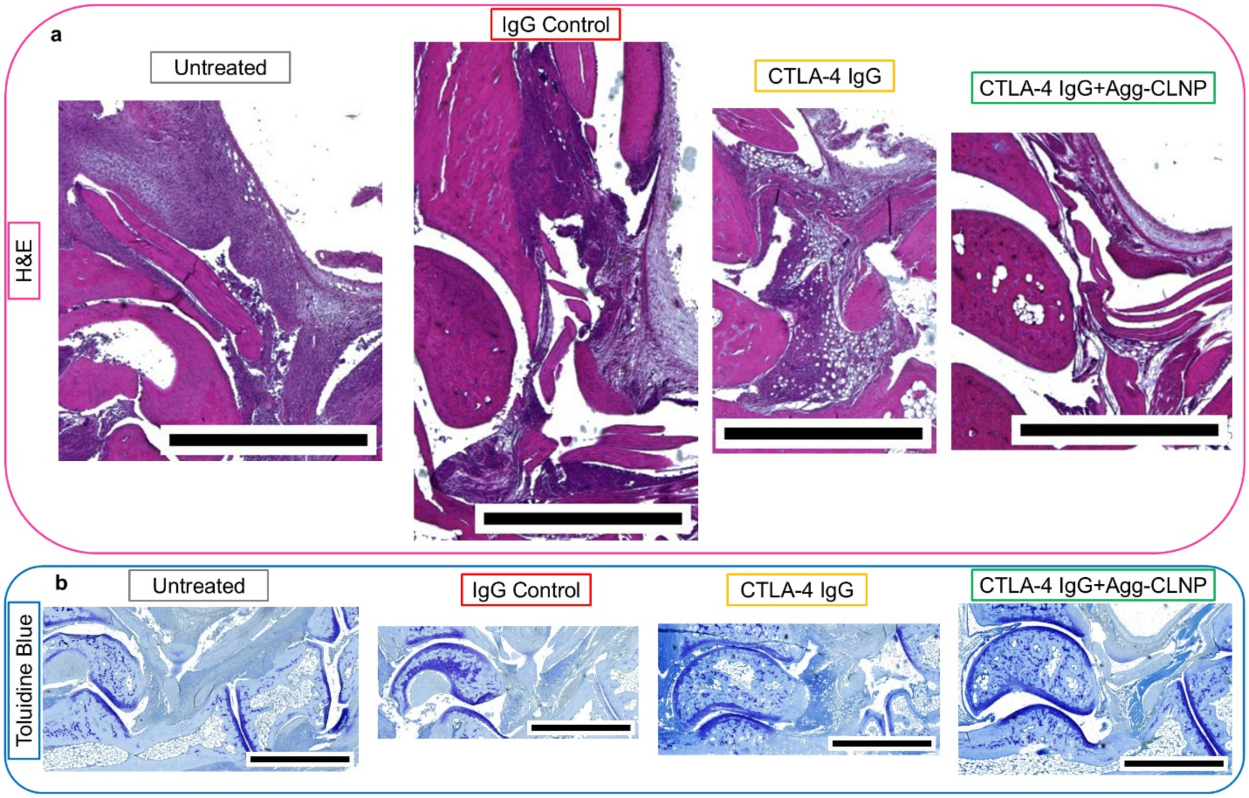

Figure S7. Magnified images of regions of interest from representative (a) H&E and (b) toluidine blue stained ankle sections in Figure 4 from untreated, IgG control, CTLA-4, and CTLA-4+Agg-CLNP treated mice. Scale bar is 1 mm.

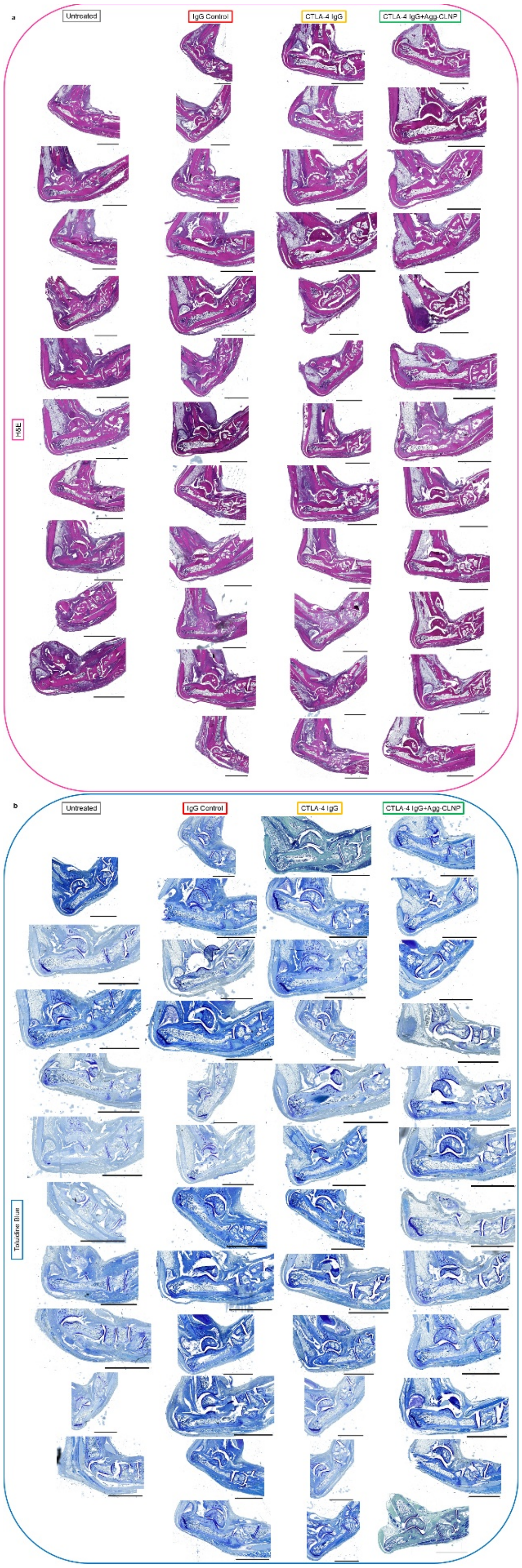

Figure S8. (a) H&E and (b) toluidine blue sections used in Figure 4. Scale bar is 2 mm.

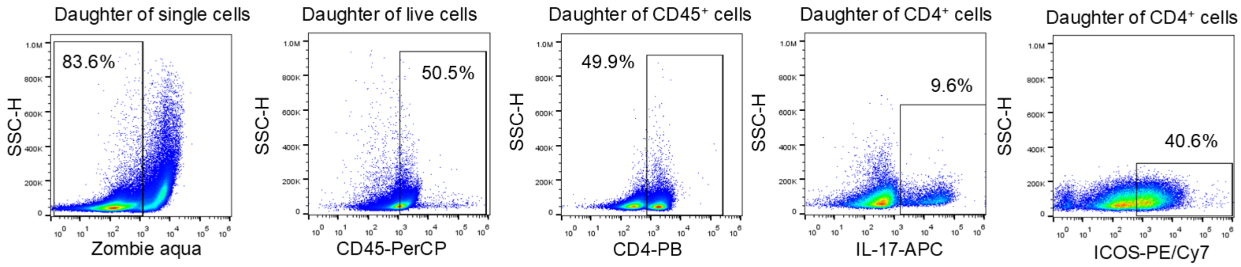

Figure S9. Flow gating strategy of CD4⁺ T cells from tissues of SKG mice.

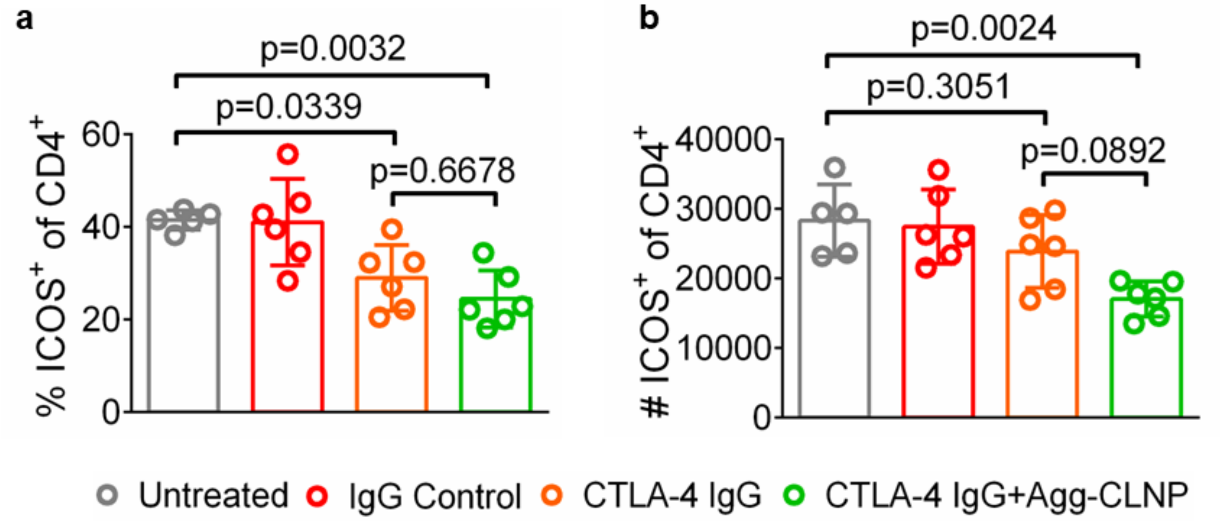

Figure S10. CTLA-4 IgG+Agg-CLNP reduces ICOS$^+$CD4$^+$ T cells in lymph nodes of SKG mice. (a) Percent of ICOS positive CD4+ T cells from the popliteal and inguinal lymph nodes. (b) Number of ICOS positive CD4+ T cells from the popliteal and inguinal lymph nodes. Data in (a-b) are means±SD of five experimental replicates for untreated group and 6 experimental replicates for all other groups. Statistical analyses in (a-b) were performed using ANOVA with Tukey's multiple comparison test.

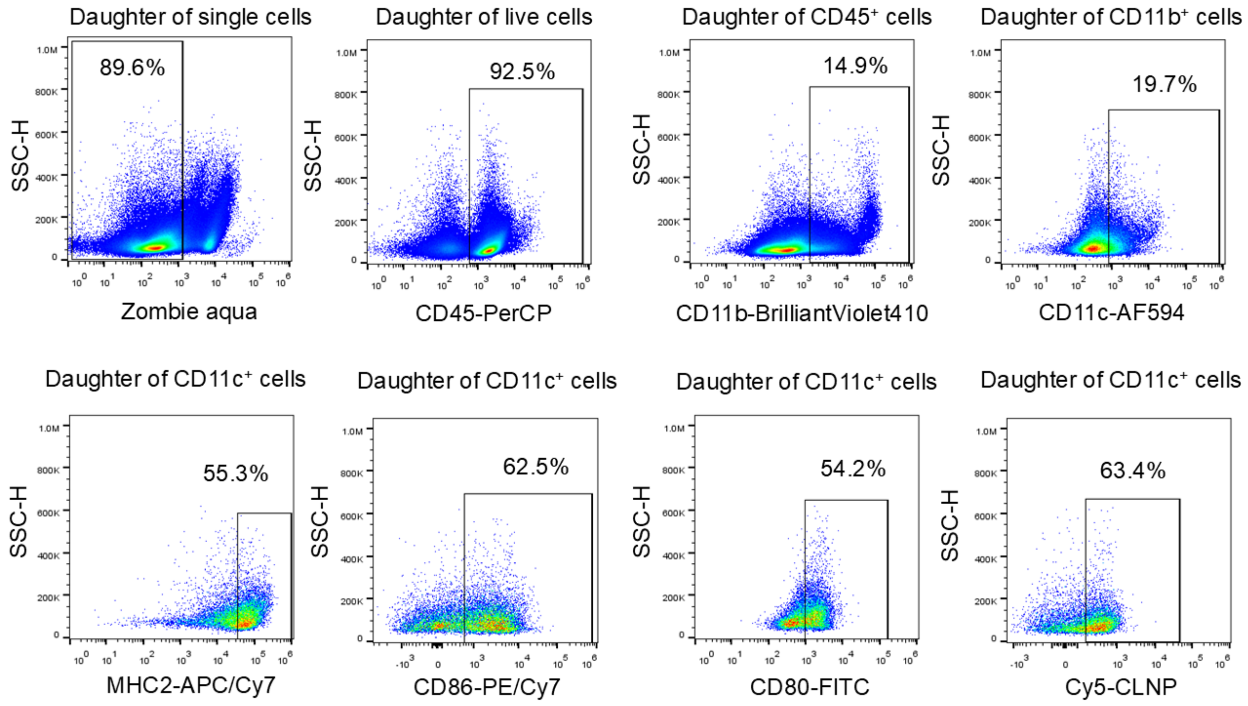

Figure S11. Flow gating strategy of DC from lymph nodes of SKG mice.

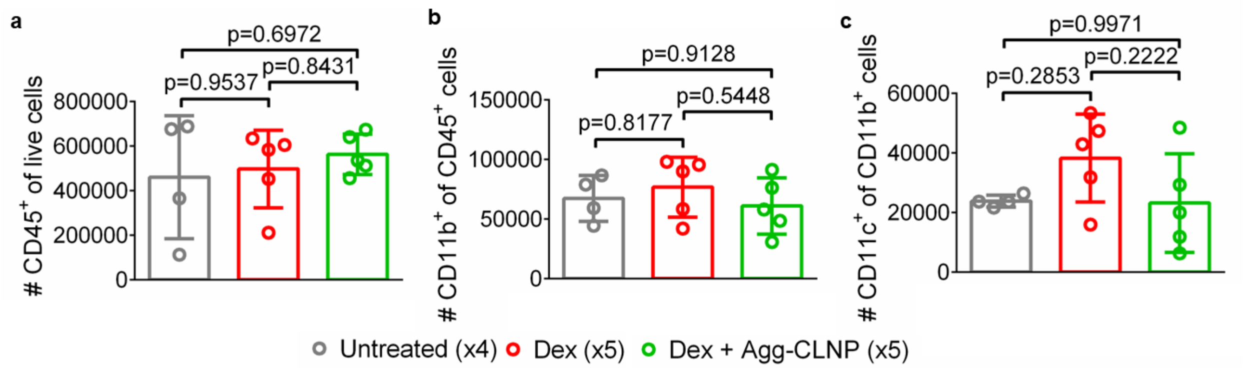

Figure S12. Agg-CLNP post dexamethasone does not significantly modulate immune cell counts in lymph nodes. Immune cell counts of (a) $CD45^+$, (b) $CD45^+CD11b^+$, and (c) $CD45^+CD11b^+CD11c^+$ from the popliteal and inguinal lymph nodes. Data in (a-c) are means±SD. Statistical analyses were performed using (a-c) ANOVA with Tukey's multiple comparison test.

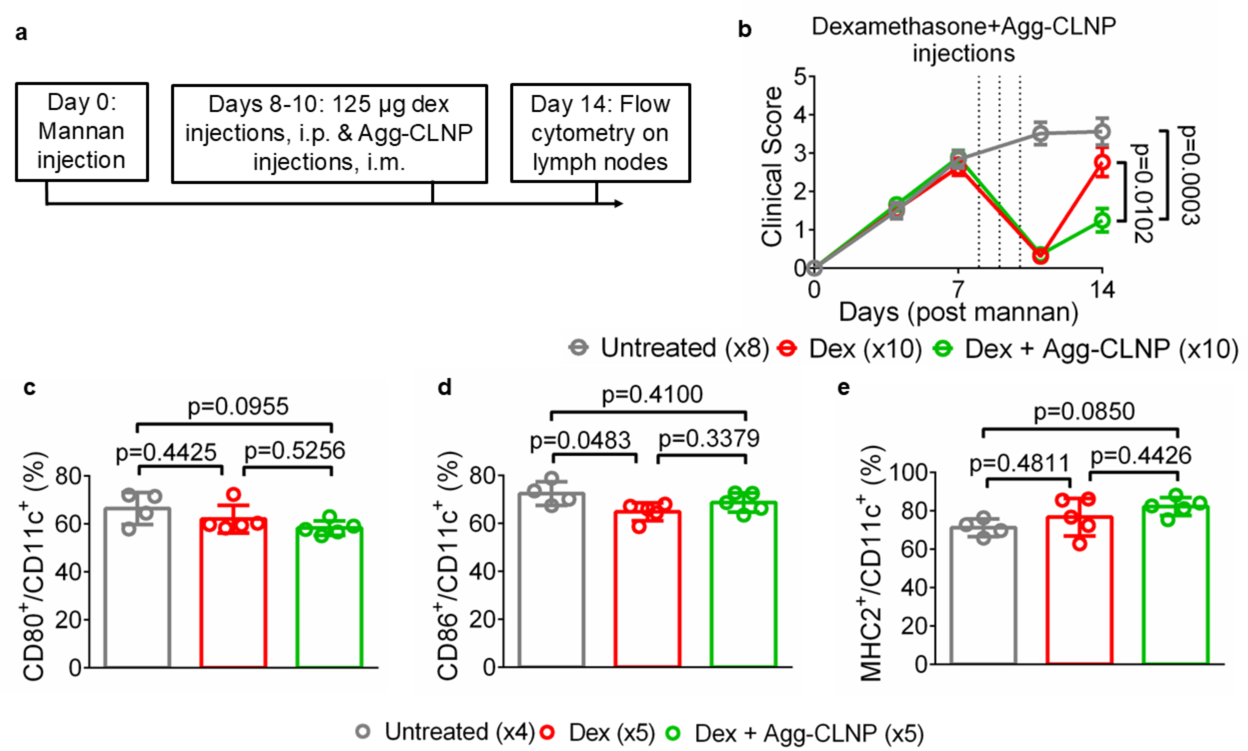

Figure S13. Co-administration of Agg-CLNP with dexamethasone is less efficacious than Agg-CLNP administration post dexamethasone. (a) Schematic and timeline of experimental procedure. (b) Clinical scores of untreated mice (n=8) and mice treated with either bolus dexamethasone (125 µg/day, n=10) or bolus dexamethasone followed by Agg-CLNP (22 µg Agg-CLNP/biceps femoris/day, n=10). (c, d, and e) Costimulatory molecules (CD80 and CD86), and MHC-2 $CD11c^+$ DC from lymph nodes on day 14 as measured by (c) CD80, (d) CD86, and (e) MHC-2. Data in (b) are means±SEM. Data in (c-e) are means±SD. Statistical analyses in (b-e) were performed using ANOVA with Tukey's multiple comparison test. Statistical analyses of (b) consisted solely of day 14 clinical scores.

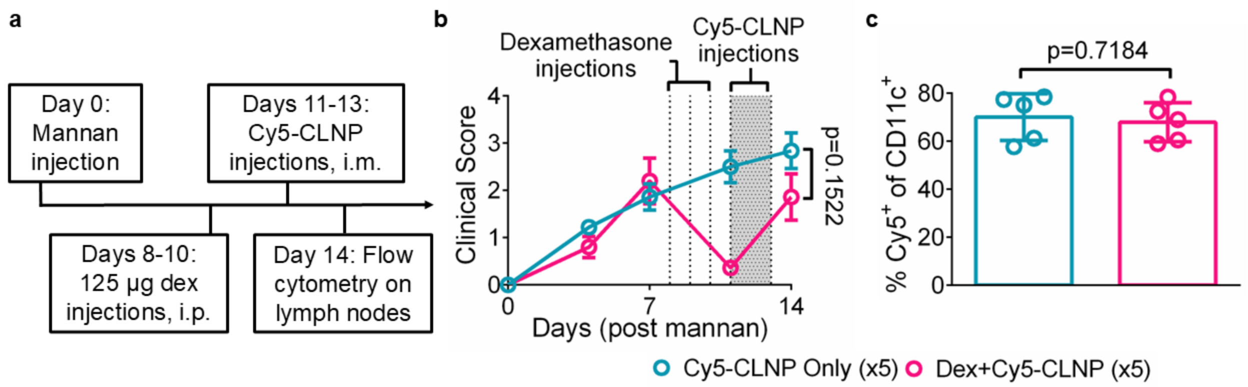

Figure S14. Dexamethasone does not affect DC ability to uptake CLNP. (a) Schematic and timeline of experimental procedure. (b) Clinical scores of Cy5-CLNP treated mice (n=5) and mice treated with dexamethasone (125 µg/day) followed by Cy5-CLNP (22 µg Cy5-CLNP/biceps femoris/day, n=5). (c) $Cy5^+$ $CD11c^+$ DC from lymph nodes on day 14. Data in (b) are means±SEM. Data in (c) are means±SD. Statistical analysis in (b) was performed using ANOVA with Tukey's multiple comparison test and in (c) was performed with unpaired Student's *t*-test with Welsch's correction. Statistical analysis of (b) consisted solely of day 14 clinical scores.

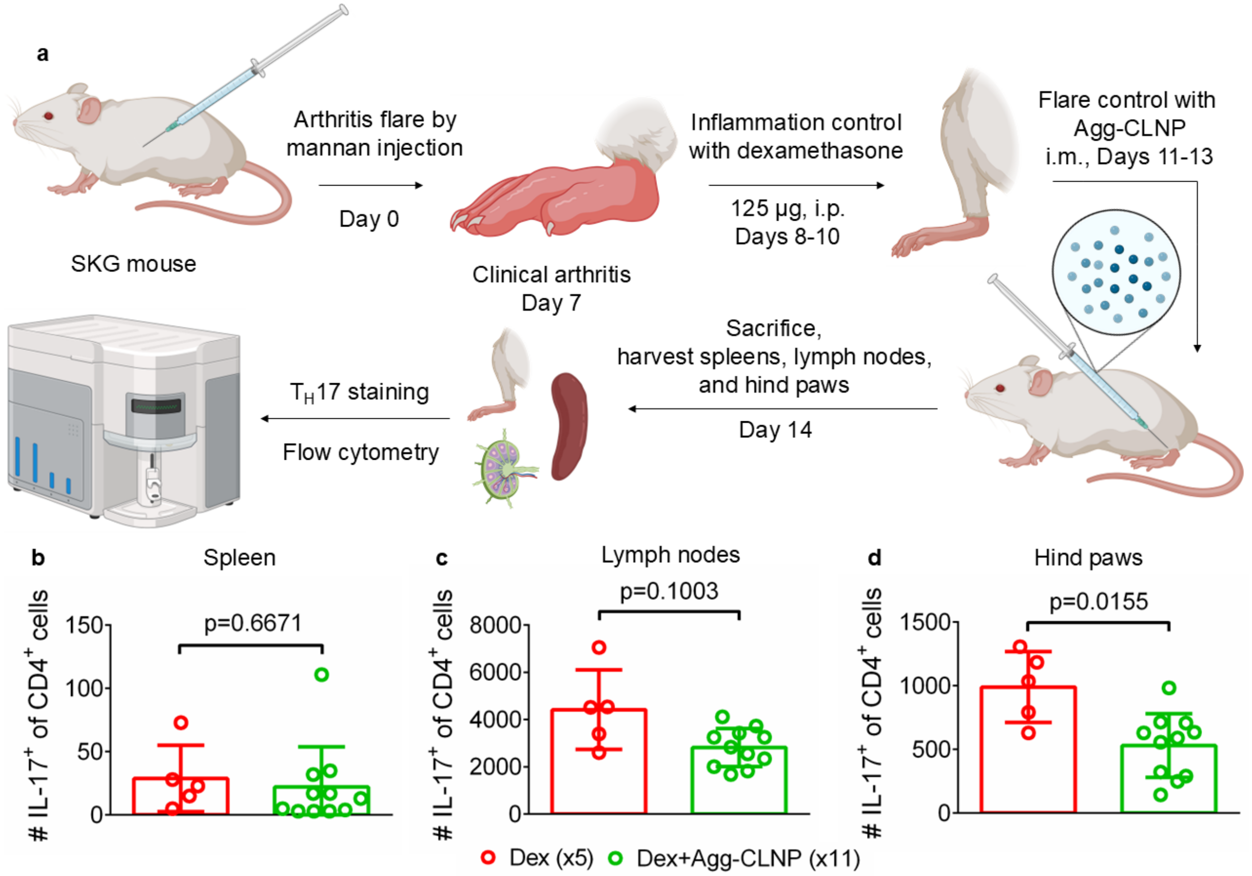

Figure S15. Agg-CLNP locally reduces pathogenic T_H17 cells in SKG mice. (a) Schematic and timeline of experimental procedure. (b) Quantification of IL-17$^+$ CD4$^+$ T_H17 cells isolated from the spleen. (c) Quantification of IL-17$^+$ CD4$^+$ T_H17 cells isolated from the inguinal and popliteal lymph nodes. (d) Quantification of IL-17$^+$ CD4$^+$ T_H17 cells isolated from the hind paws. Statistical analyses were performed using (b-d) unpaired Student's *t*-test with Welsch's correction. Data in (b-d) are means±SD. Schematic in (a) was composed using BioRender.

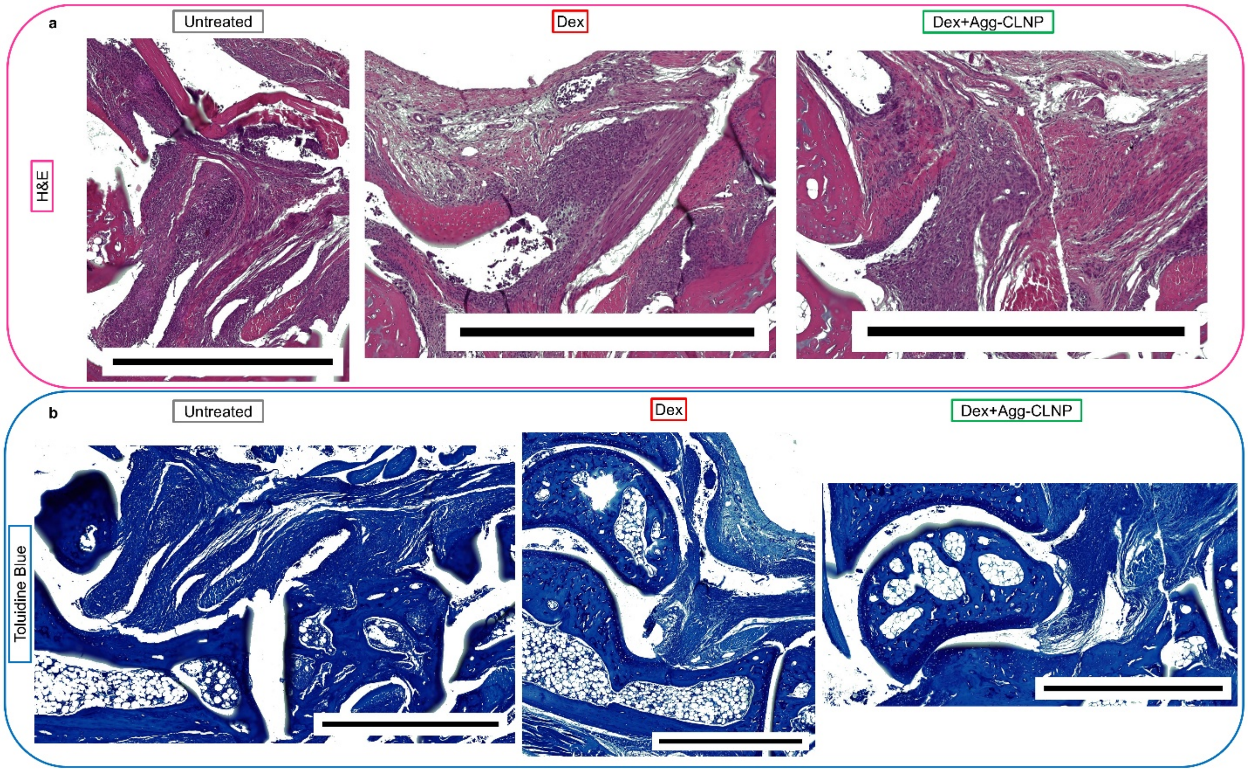

Figure S16. Magnified images of regions of interest from representative (a) H&E and (b) toluidine blue stained ankle sections in Figure 6 from untreated, dexamethasone, and Dex+Agg-CLNP treated mice. Scale bar is 1 mm.

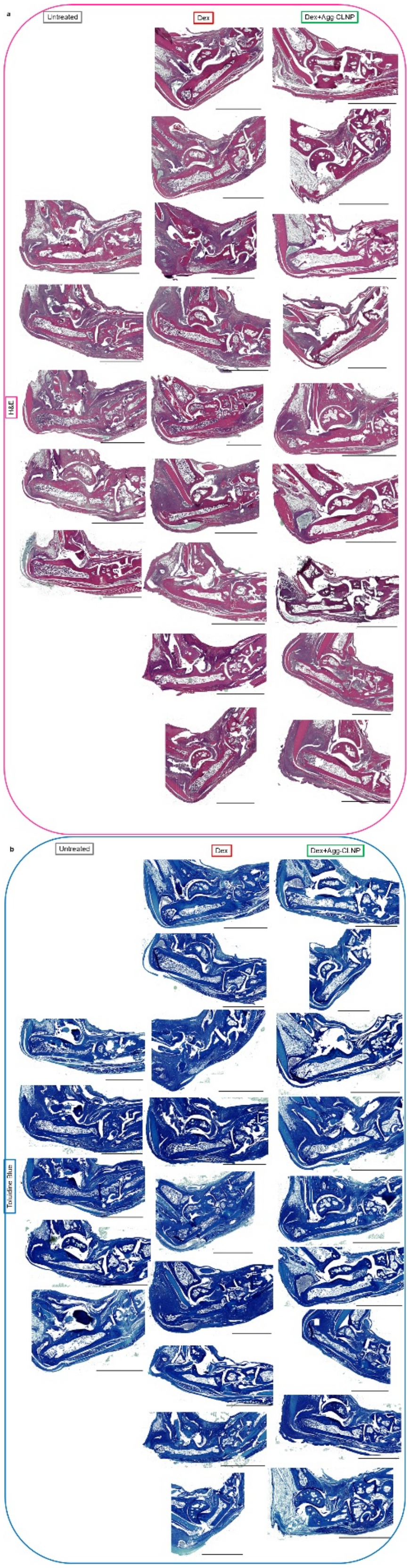

Figure S17. (a) H&E and (b) toluidine blue sections used in Figure 6. Scale bar is 2 mm.

Batch	Z-avg / nm	PDI
1	74	0.06
2	68	0.09
3	68	0.08

Table S1. DLS of three batches of Agg-CLNP.

Batch	Calcitriol concentration / ng/mL	Encapsulation efficiency / %
1	267	20.5
2	273	20.9
3	277	21.2

Table S2. Calcitriol encapsulation efficiency in Agg-CLNP.

Donor	Sex	Age	Treatment(s)	Disease state
1	F	83	mycophenolate+prednisone 5 mg/day	no swollen or tender joints, RF and anti-CCP positive; ILD
2	F	60	adalimumab	no swollen or tender joints; seronegative; erosions
3	M	68	adalimumab	minimal synovitis; anti-CCP positive
4	F	44	abatacept and leflunomide	synovitis in hands and wrists; RF and anti-CCP positive
5	F	53	entanercept	no synovitis except in one wrist; RF and anti-CCP positive
6	F	56	entanercept	no synovitis; seronegative

Table S3. RA patient donor characteristics.
